# Supplementary material for: Weakly migratory metastatic breast cancer cells activate fibroblasts via microvesicle-Tg2 to facilitate dissemination and metastasis
Source: eLife. 2022 Dec 7;11:e74433. doi: 10.7554/eLife.74433 (PMC9767463; doi:10.7554/eLife.74433)
Supplement: Source data 1. [file elife-74433-data1.zip › Western Blots/Blot Labeling.pptx]

## Slide 1
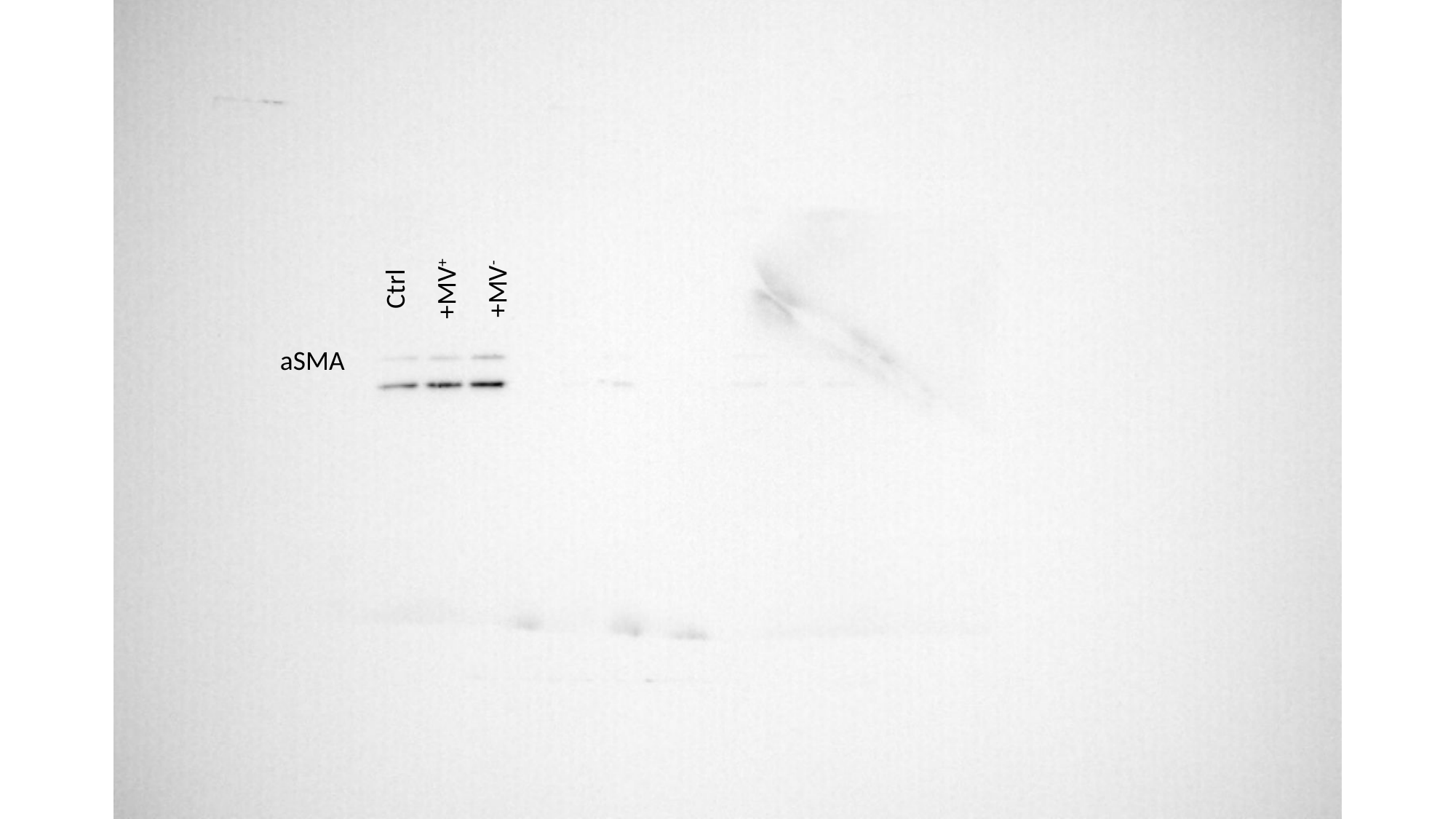

Ctrl
+MV+
+MV-
aSMA

## Slide 2
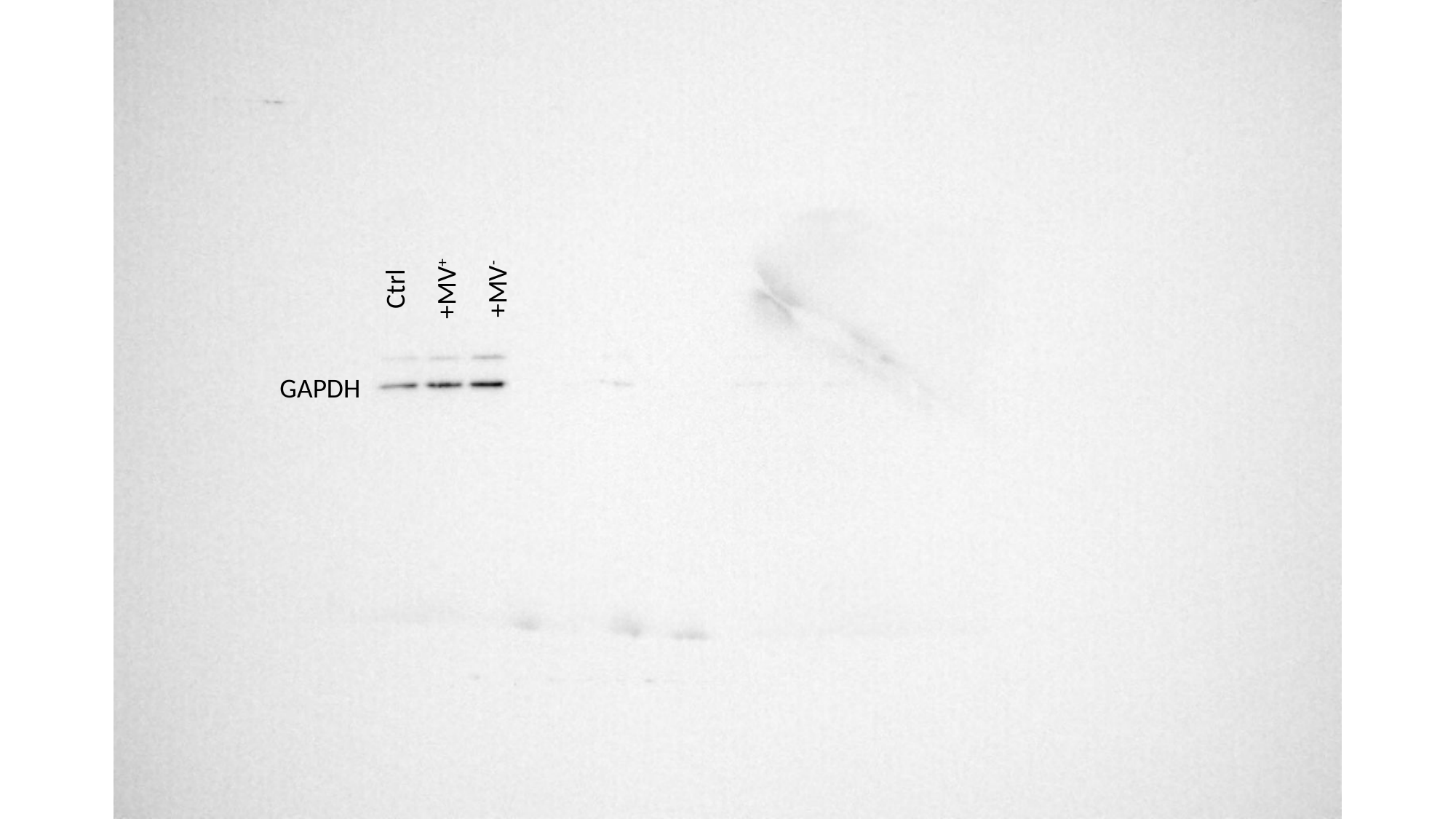

Ctrl
+MV+
+MV-
GAPDH

## Slide 3
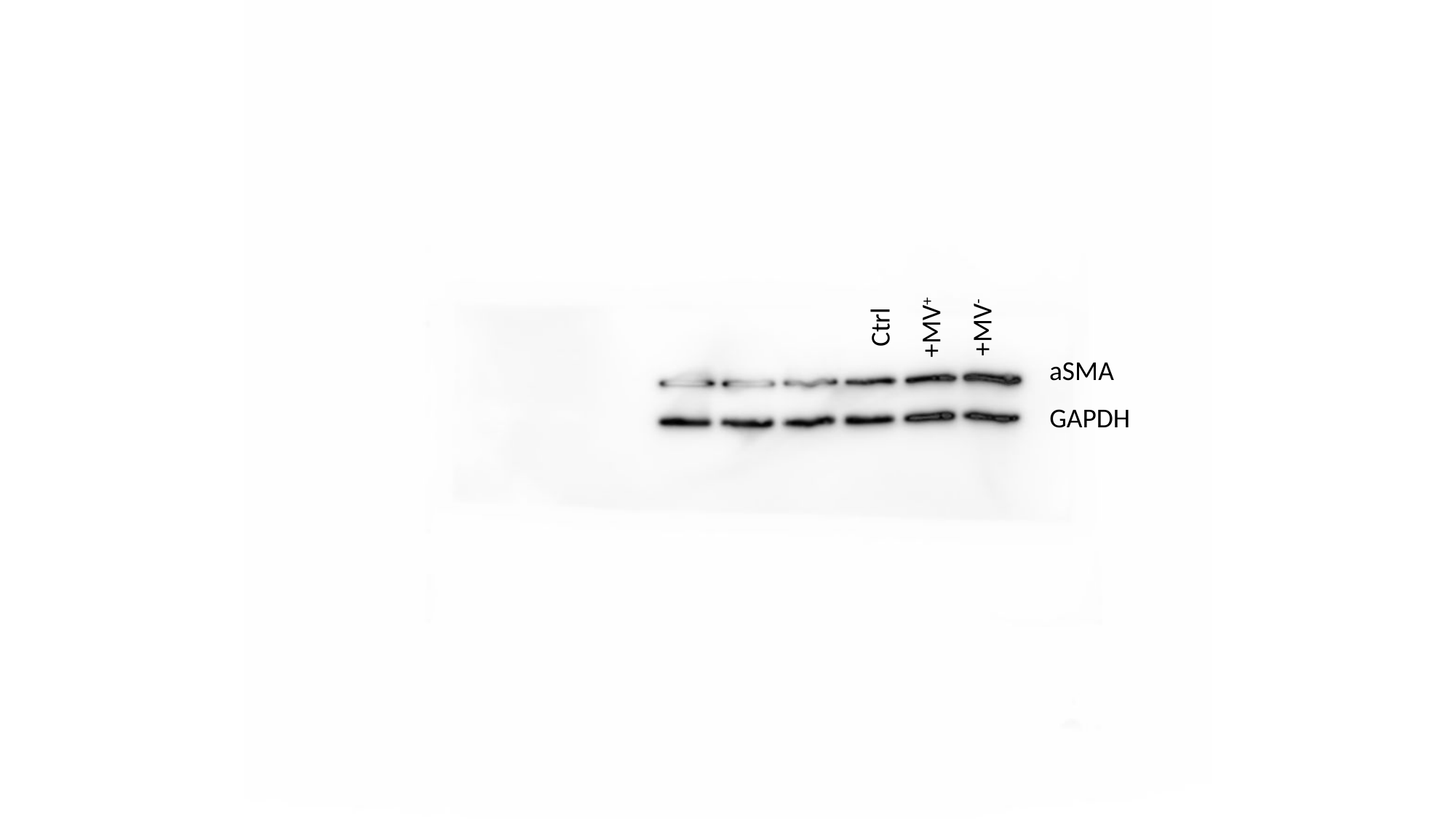

Ctrl
+MV+
+MV-
aSMA
GAPDH

## Slide 4
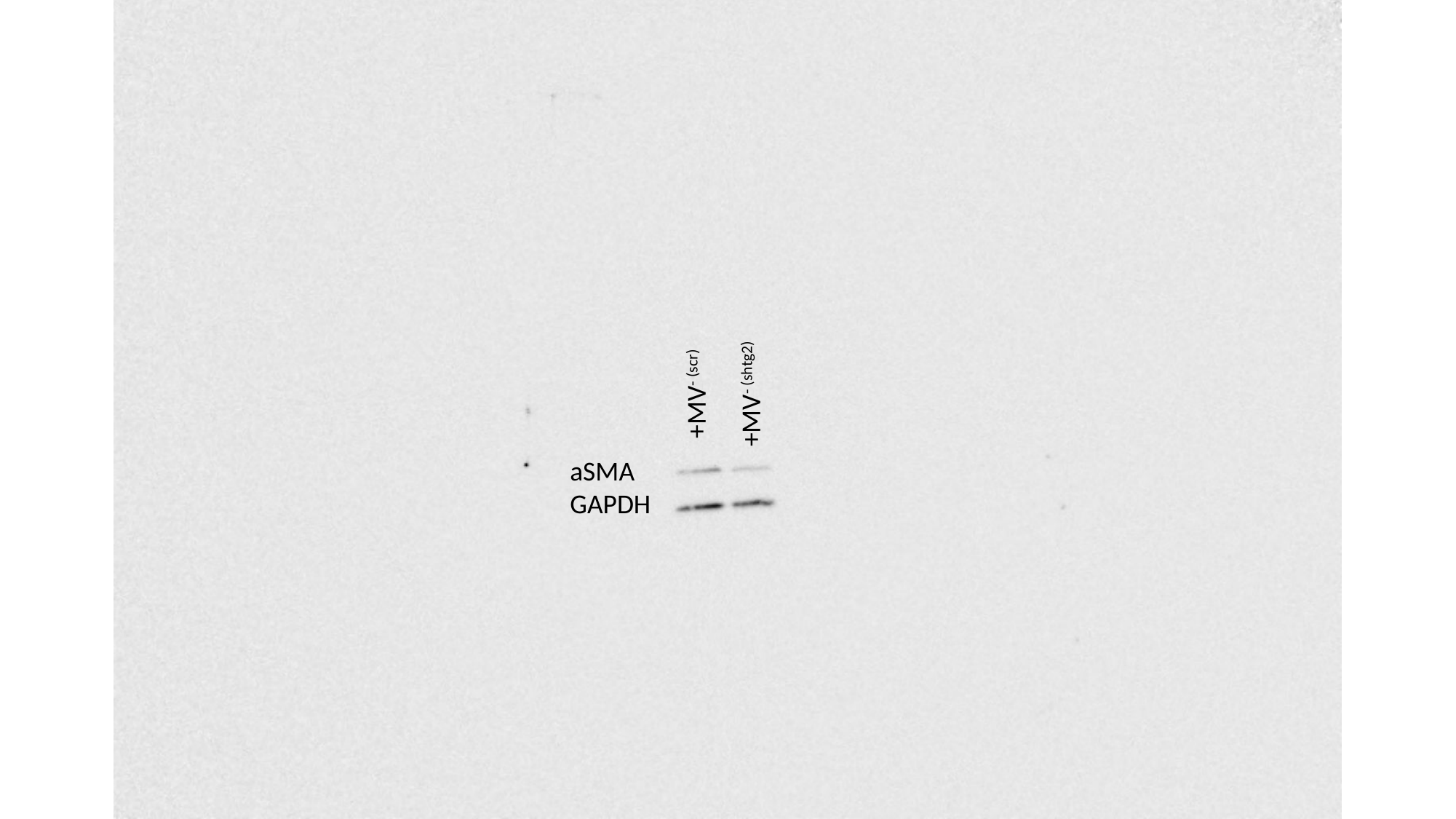

+MV- (scr)
+MV- (shtg2)
aSMA
GAPDH

## Slide 5
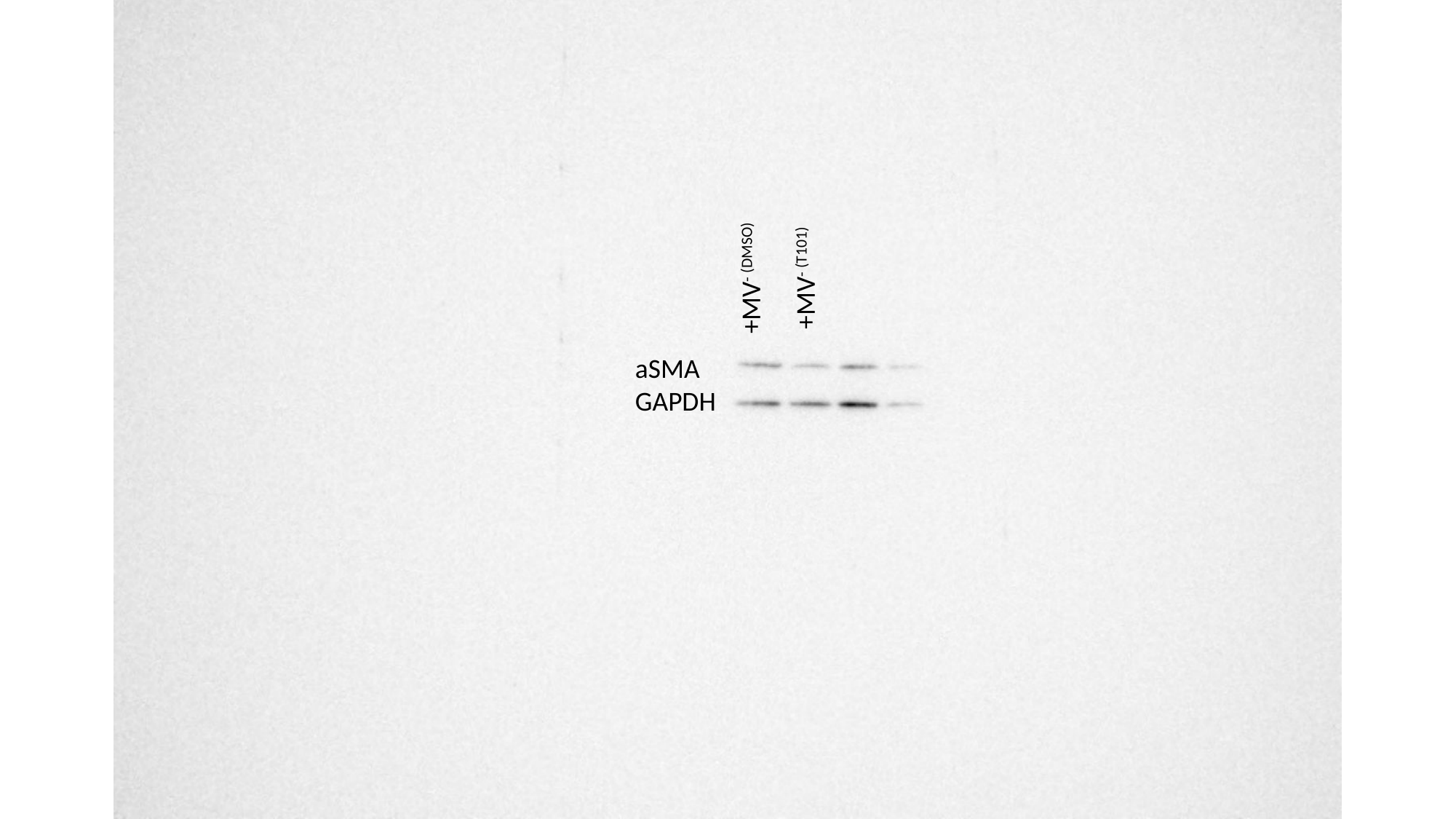

+MV- (DMSO)
+MV- (T101)
aSMA
GAPDH

## Slide 6
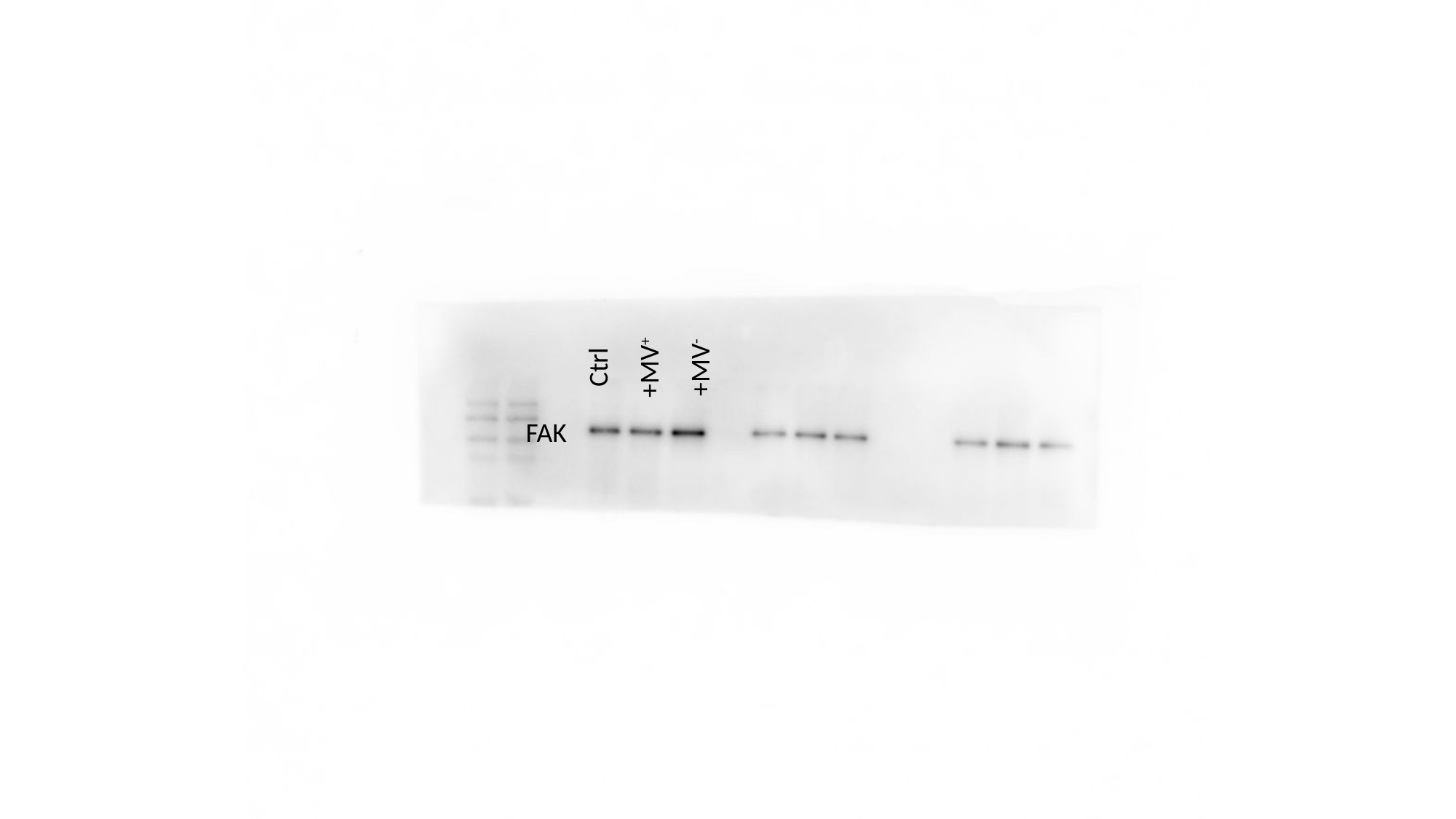

Ctrl
+MV+
+MV-
FAK

## Slide 7
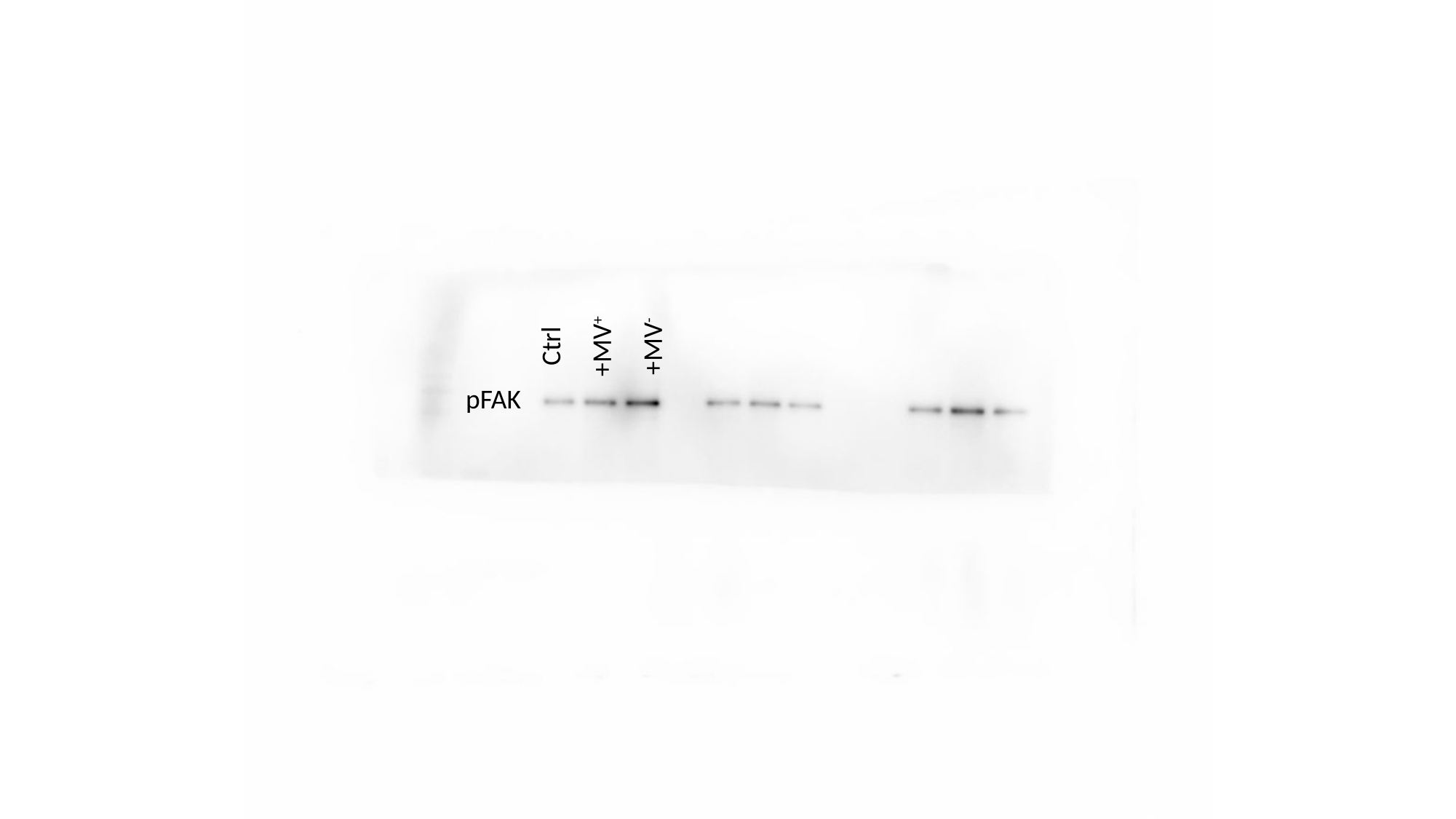

Ctrl
+MV+
+MV-
pFAK

## Slide 8
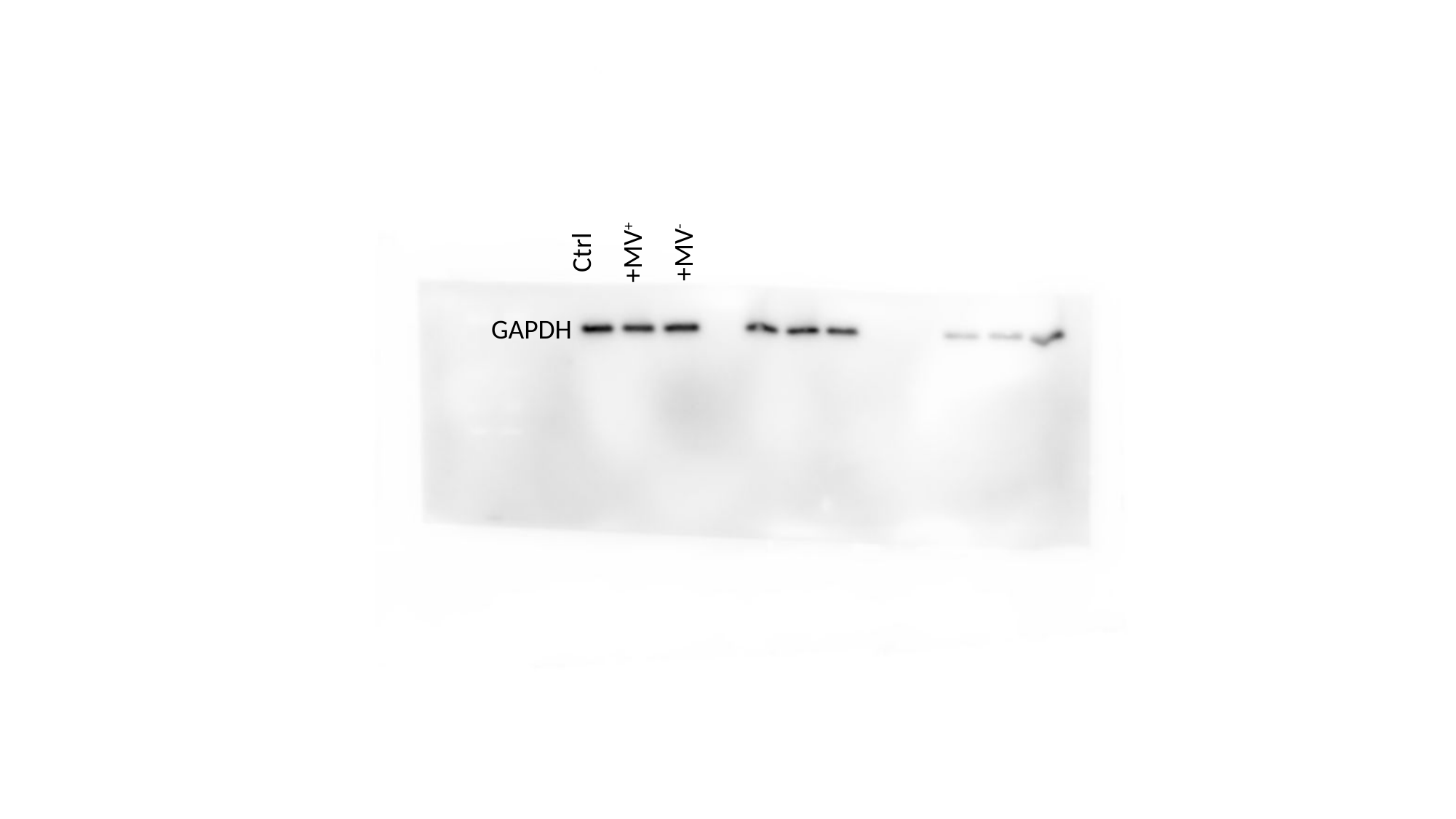

Ctrl
+MV+
+MV-
GAPDH

## Slide 9
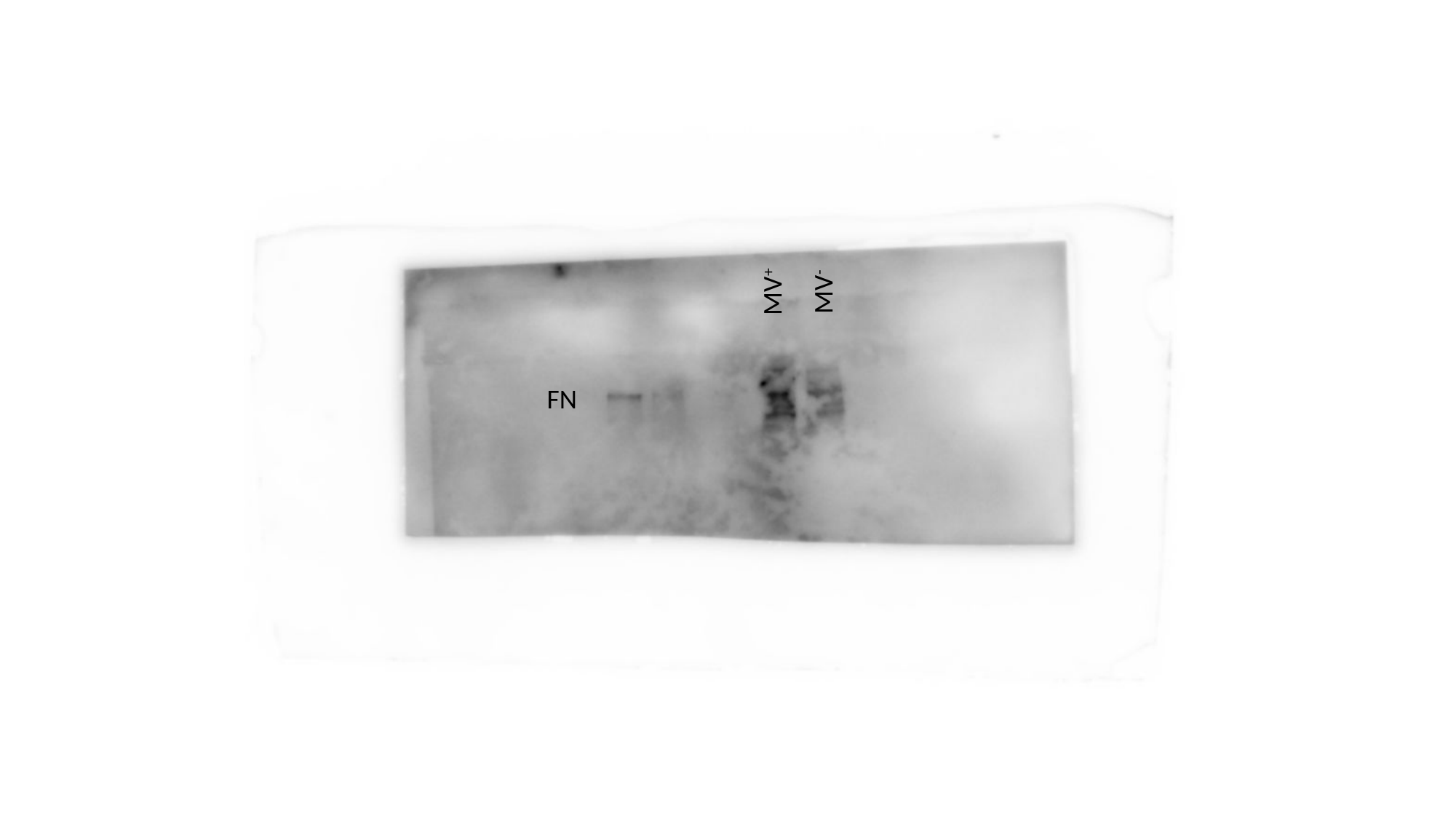

MV+
MV-
FN

## Slide 10
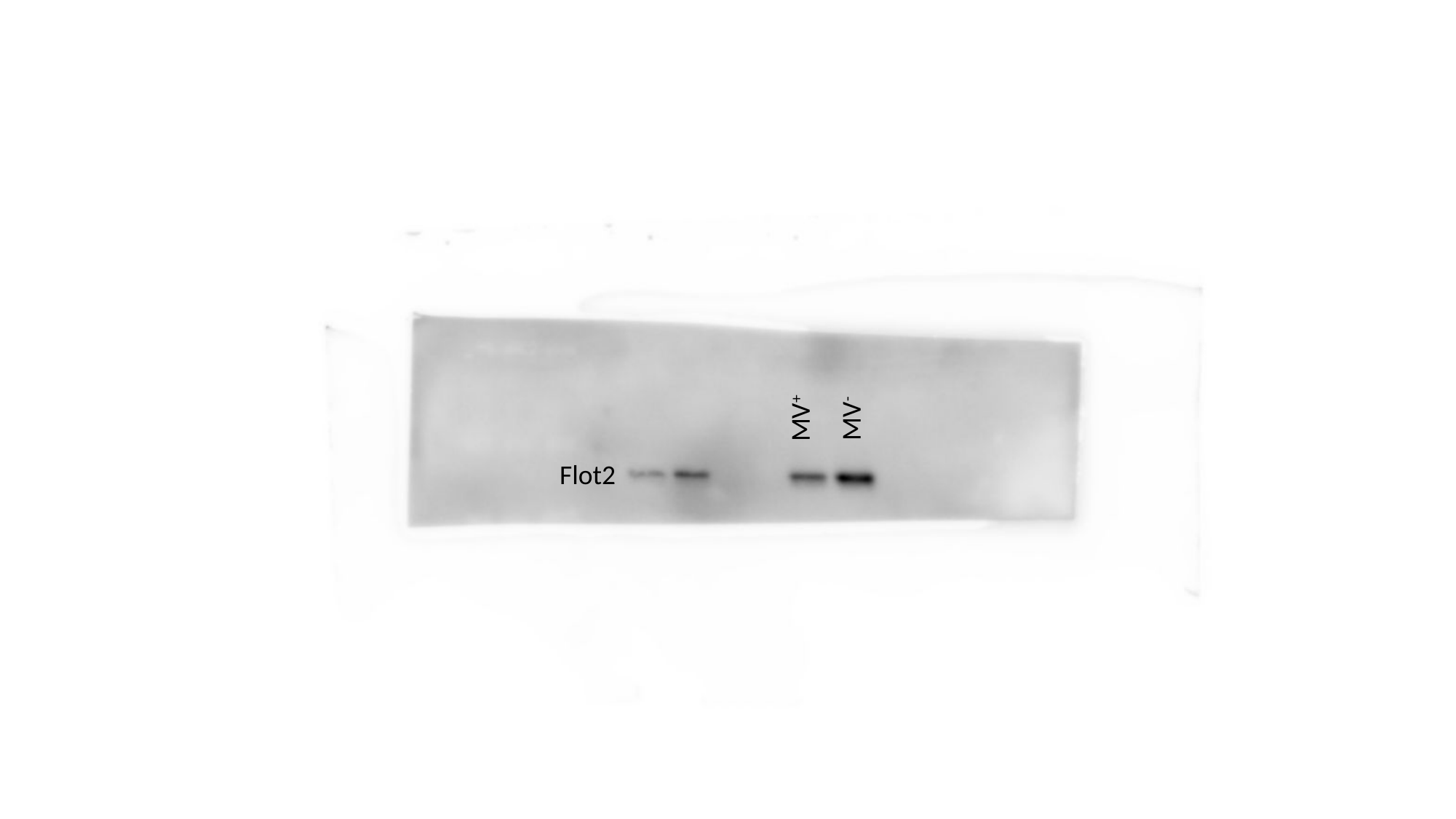

MV+
MV-
Flot2

## Slide 11
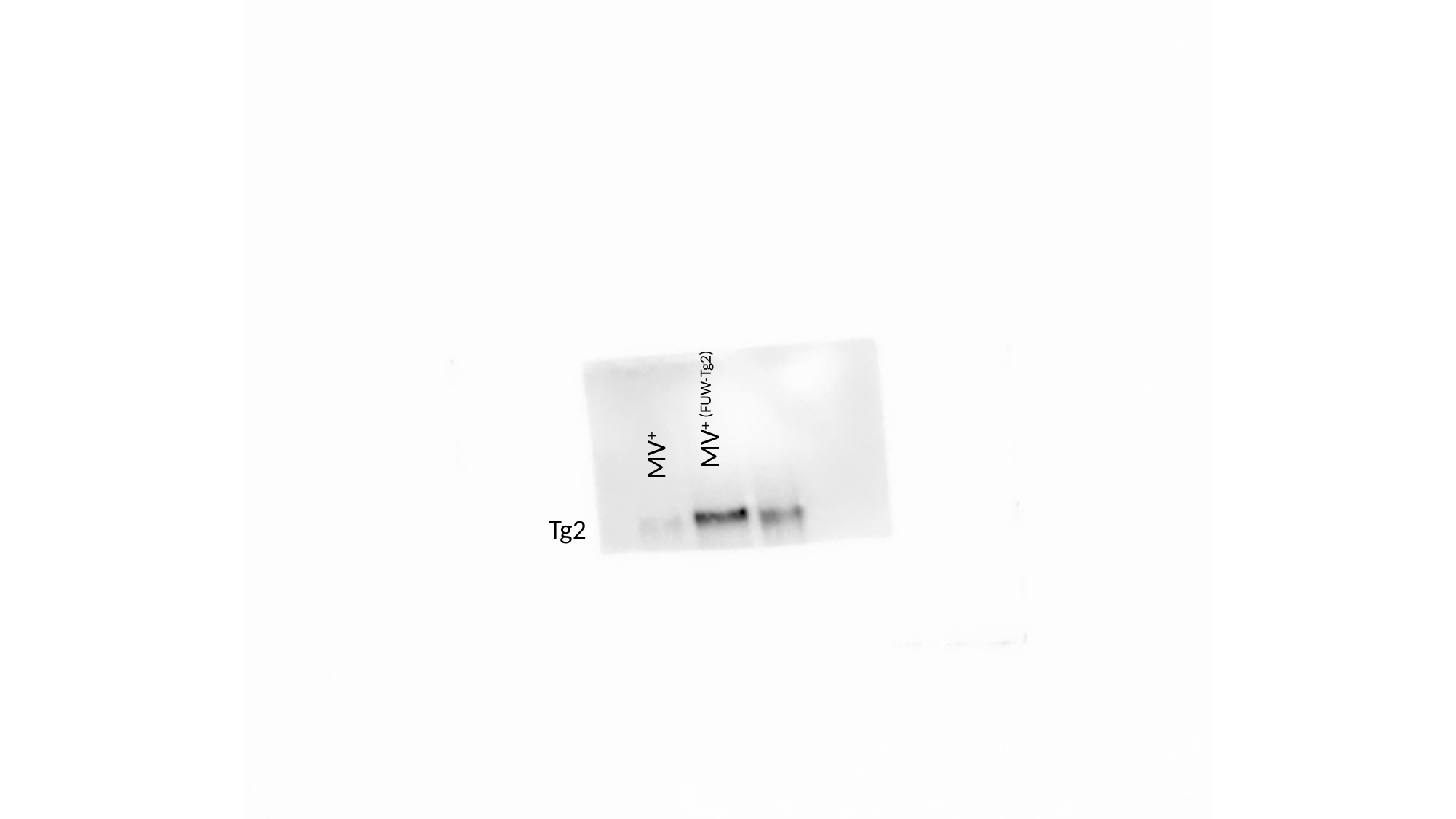

MV+ (FUW-Tg2)
MV+
Tg2

## Slide 12
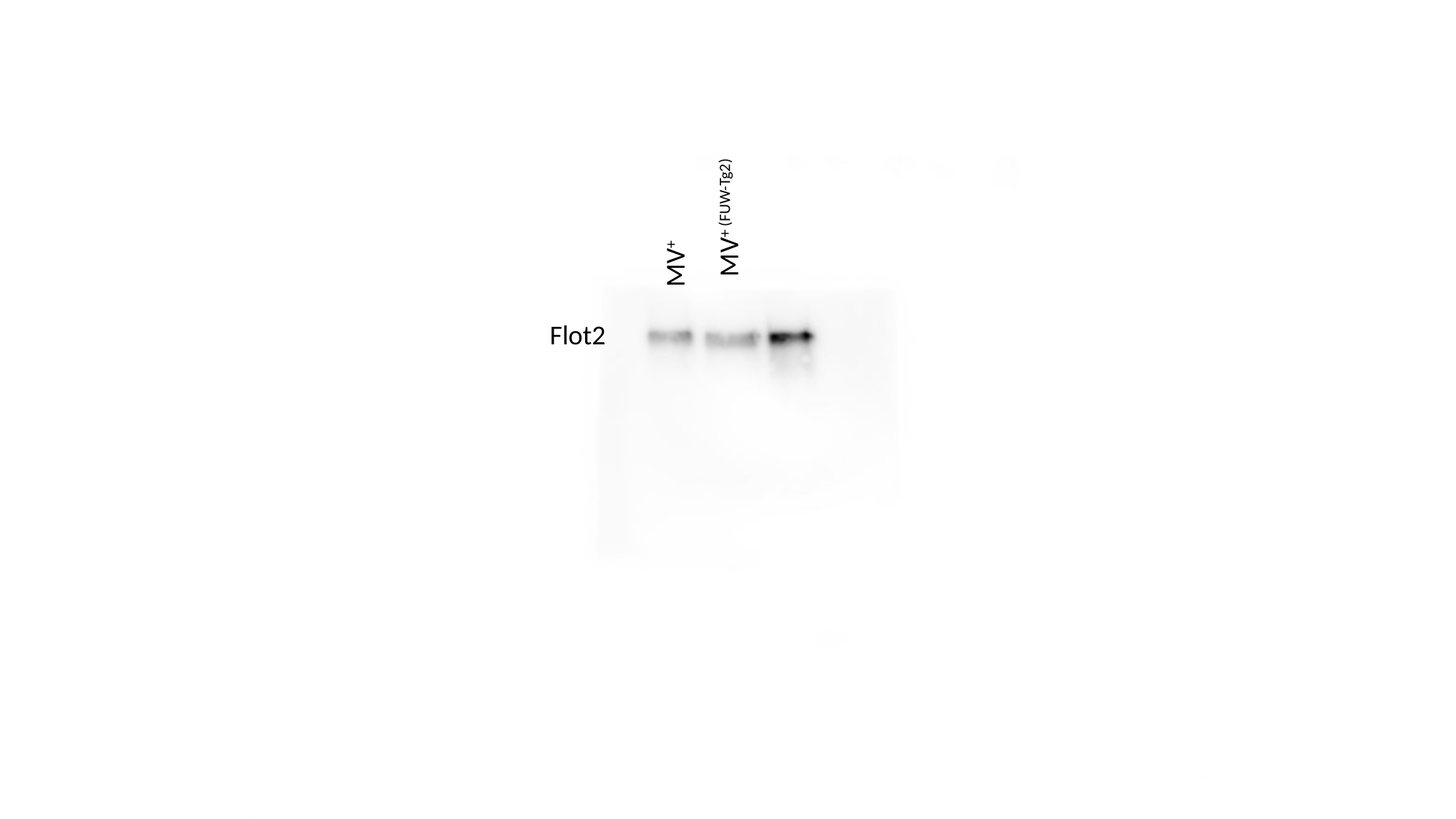

MV+ (FUW-Tg2)
MV+
Flot2

## Slide 13
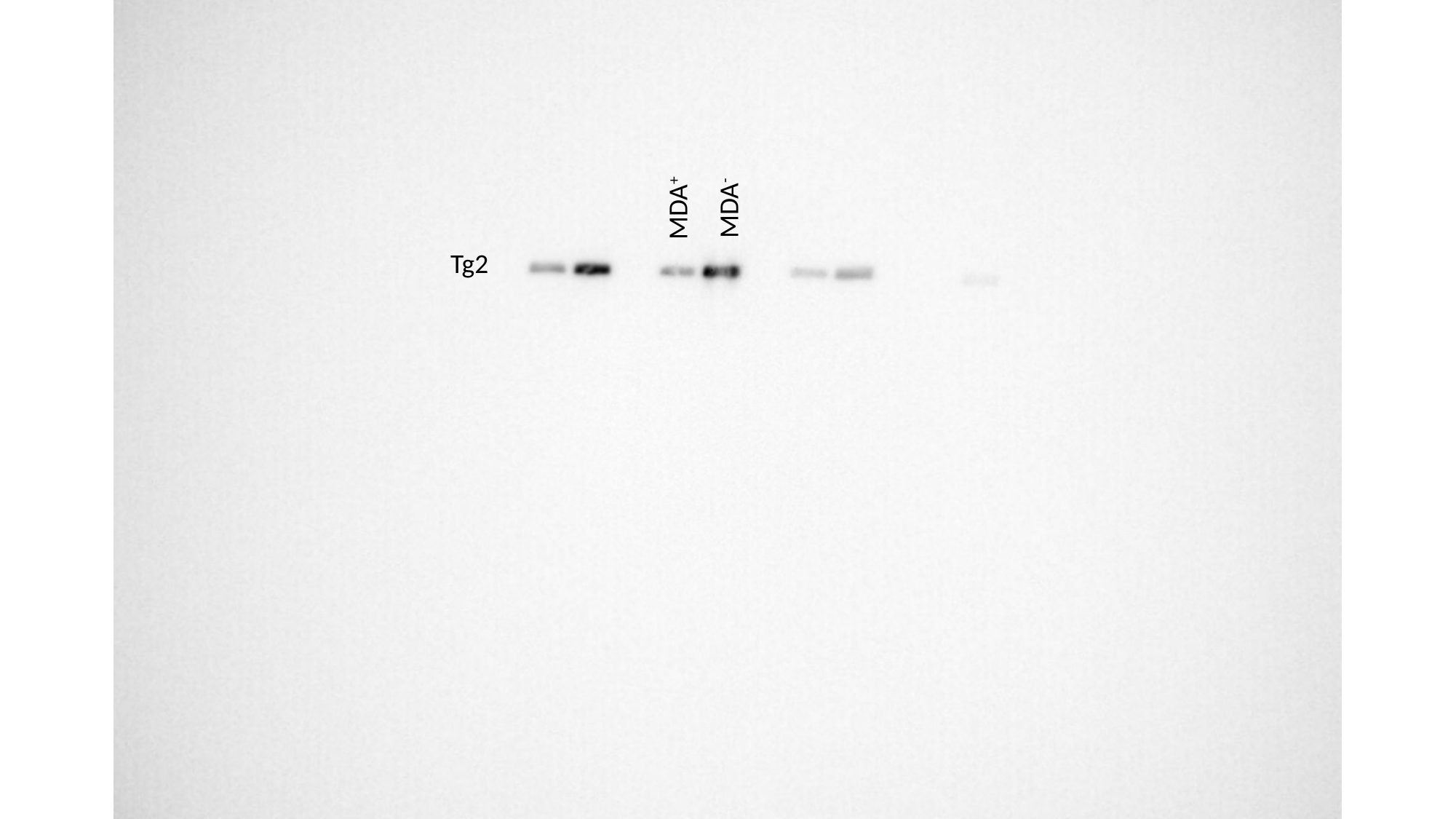

MDA+
MDA-
Tg2

## Slide 14
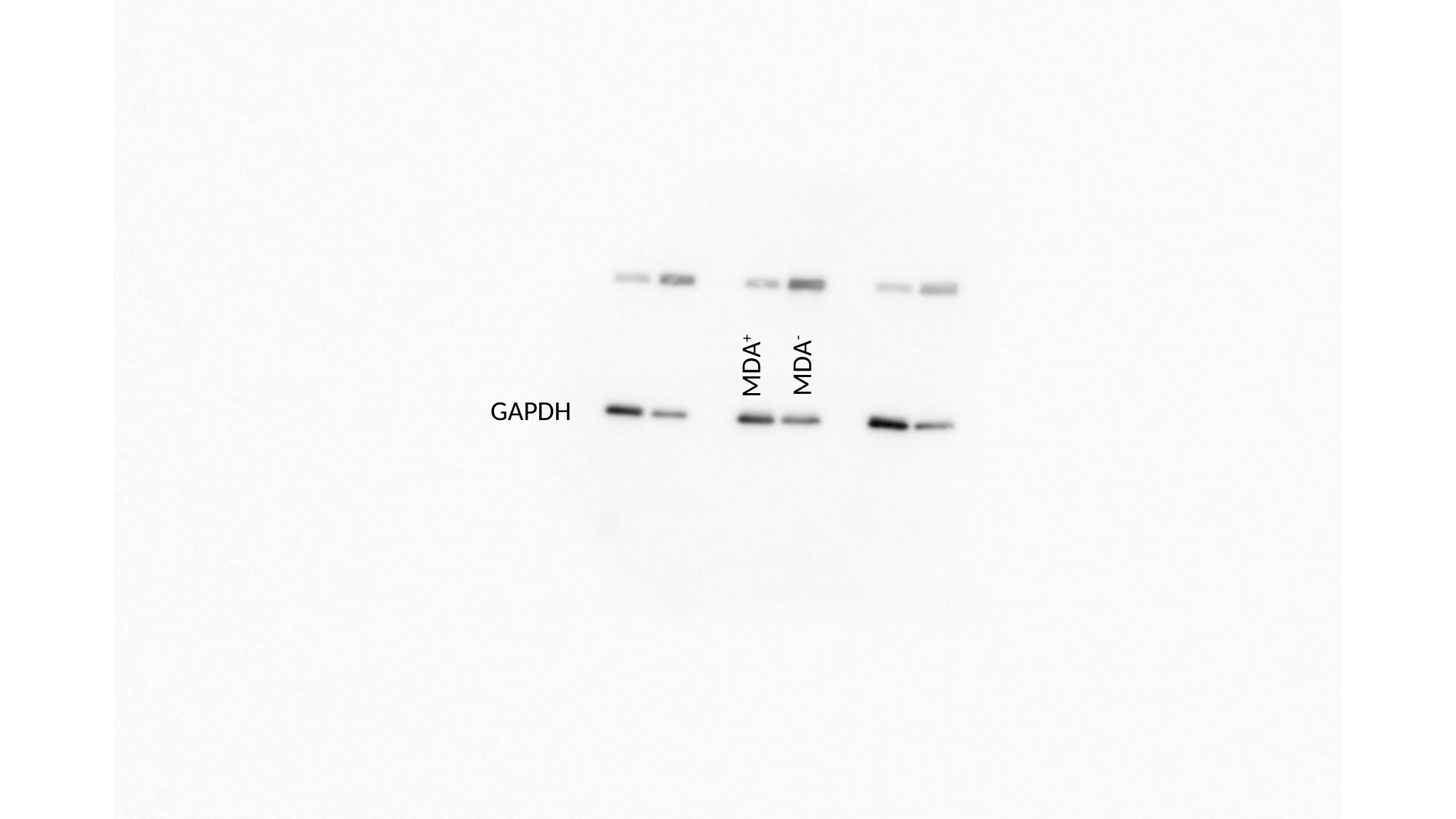

MDA+
MDA-
GAPDH

## Slide 15
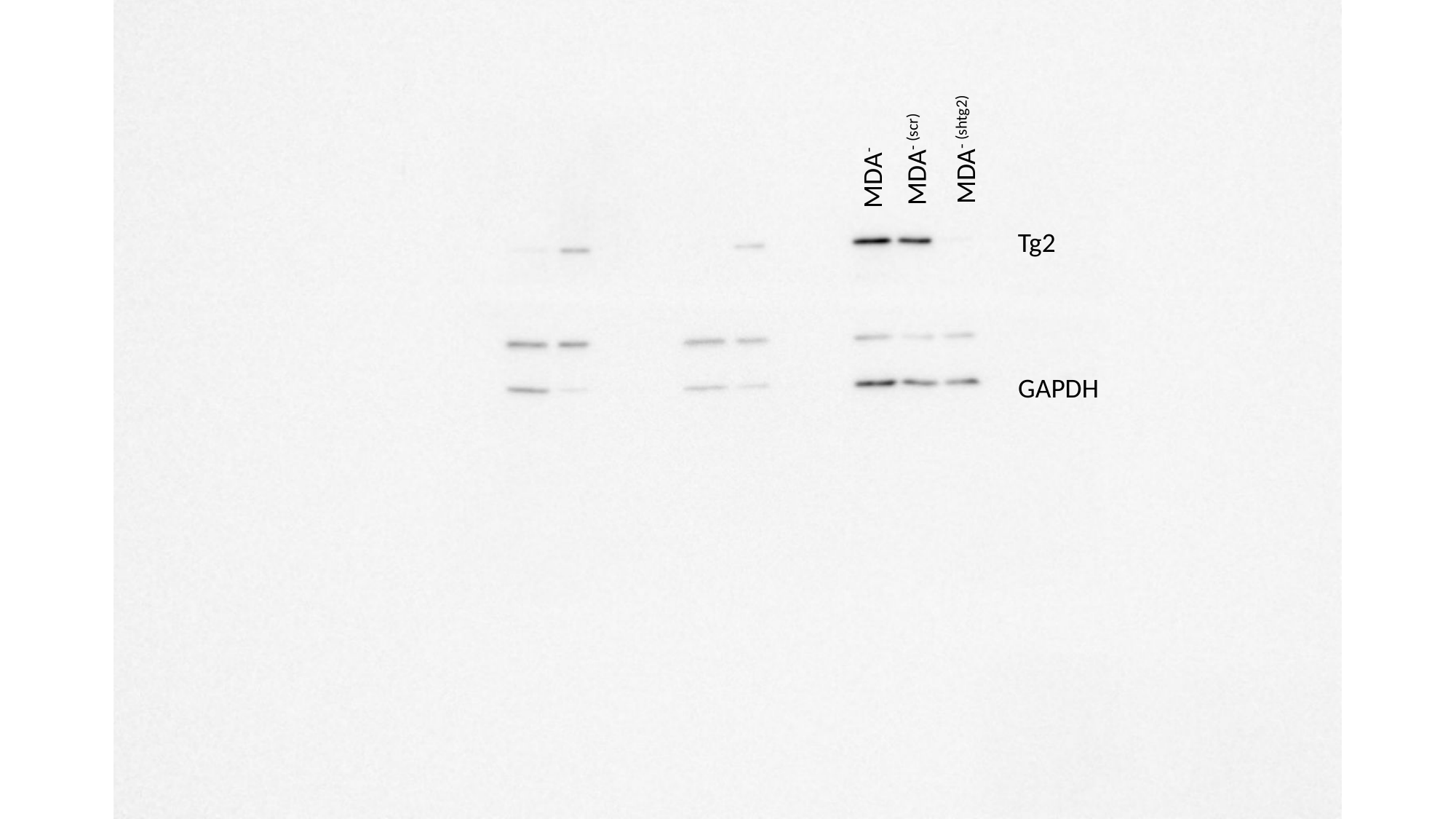

MDA- (shtg2)
MDA- (scr)
MDA-
Tg2
GAPDH

## Slide 16
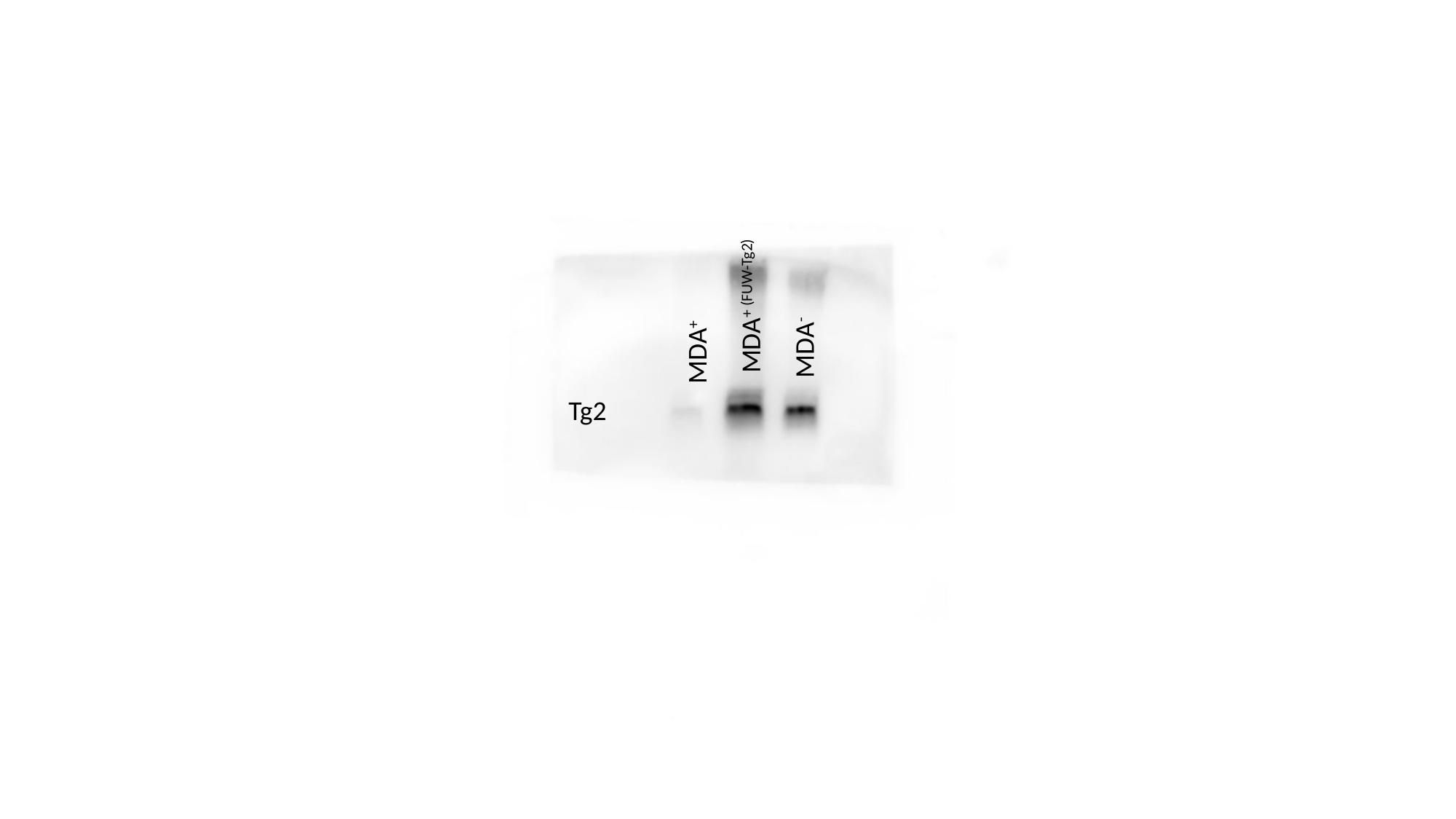

MDA+ (FUW-Tg2)
MDA-
MDA+
Tg2

## Slide 17
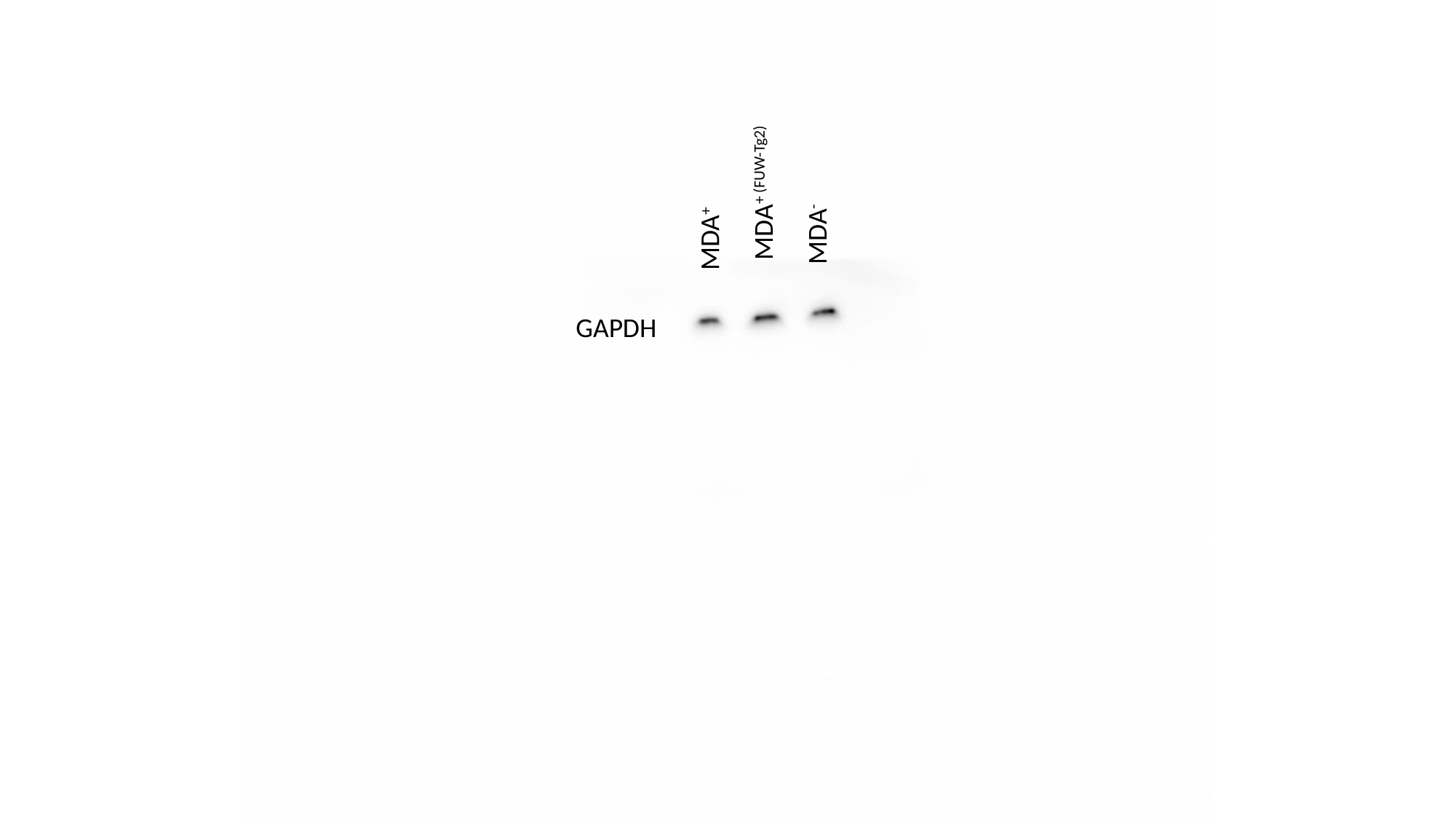

MDA+ (FUW-Tg2)
MDA-
MDA+
GAPDH

## Slide 18
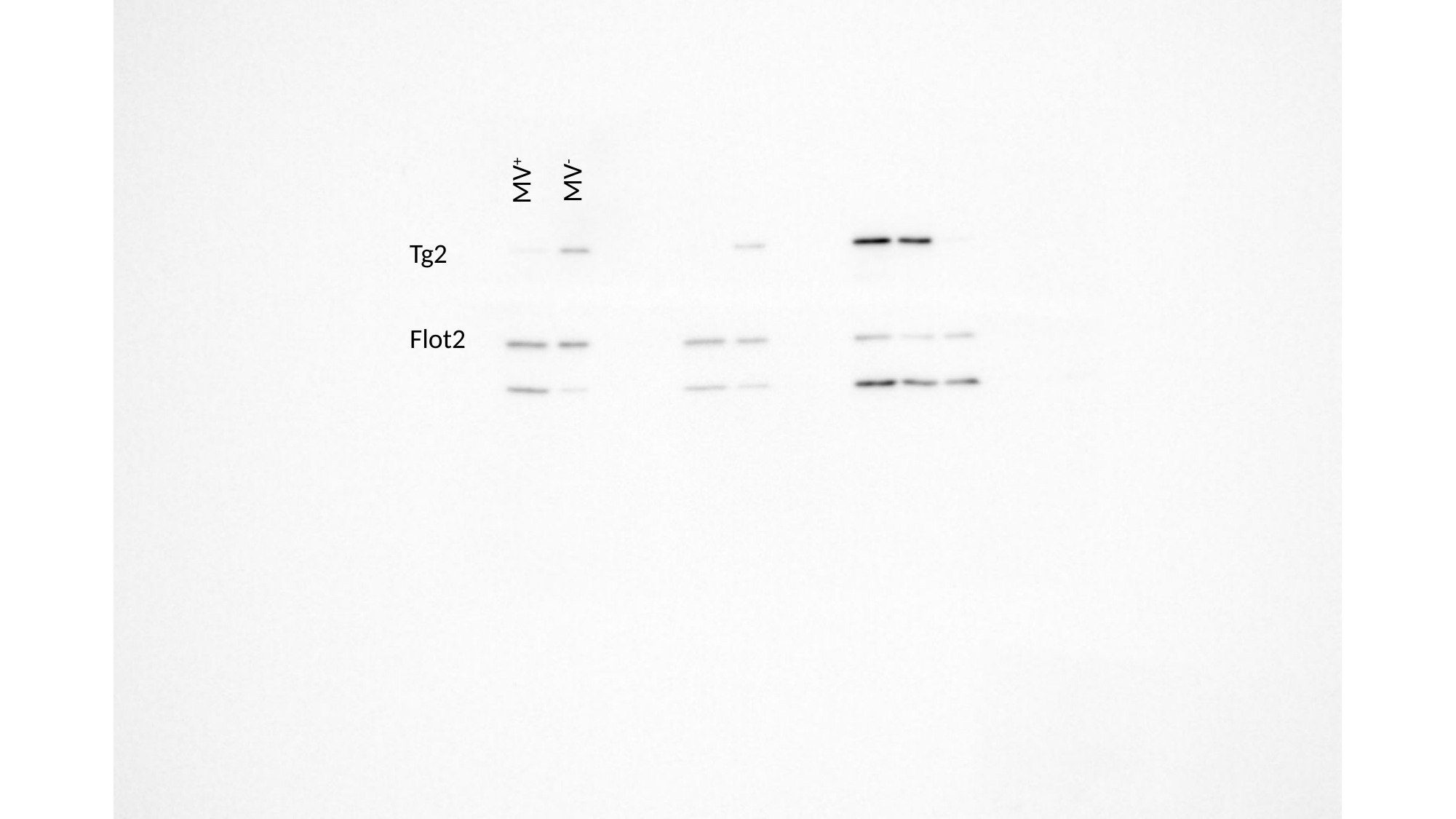

MV+
MV-
Tg2
Flot2

## Slide 19
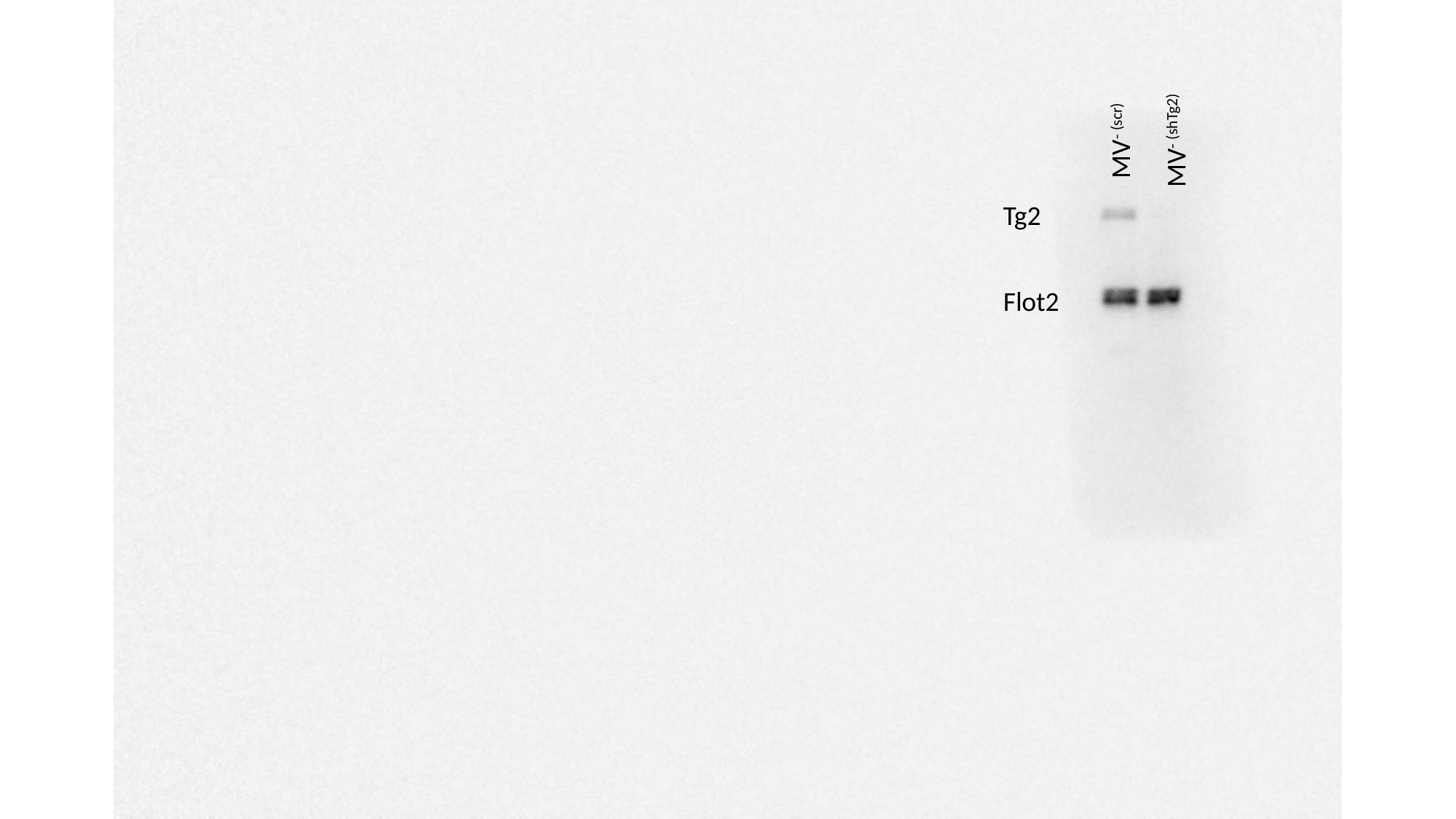

MV- (scr)
MV- (shTg2)
Tg2
Flot2

## Slide 20
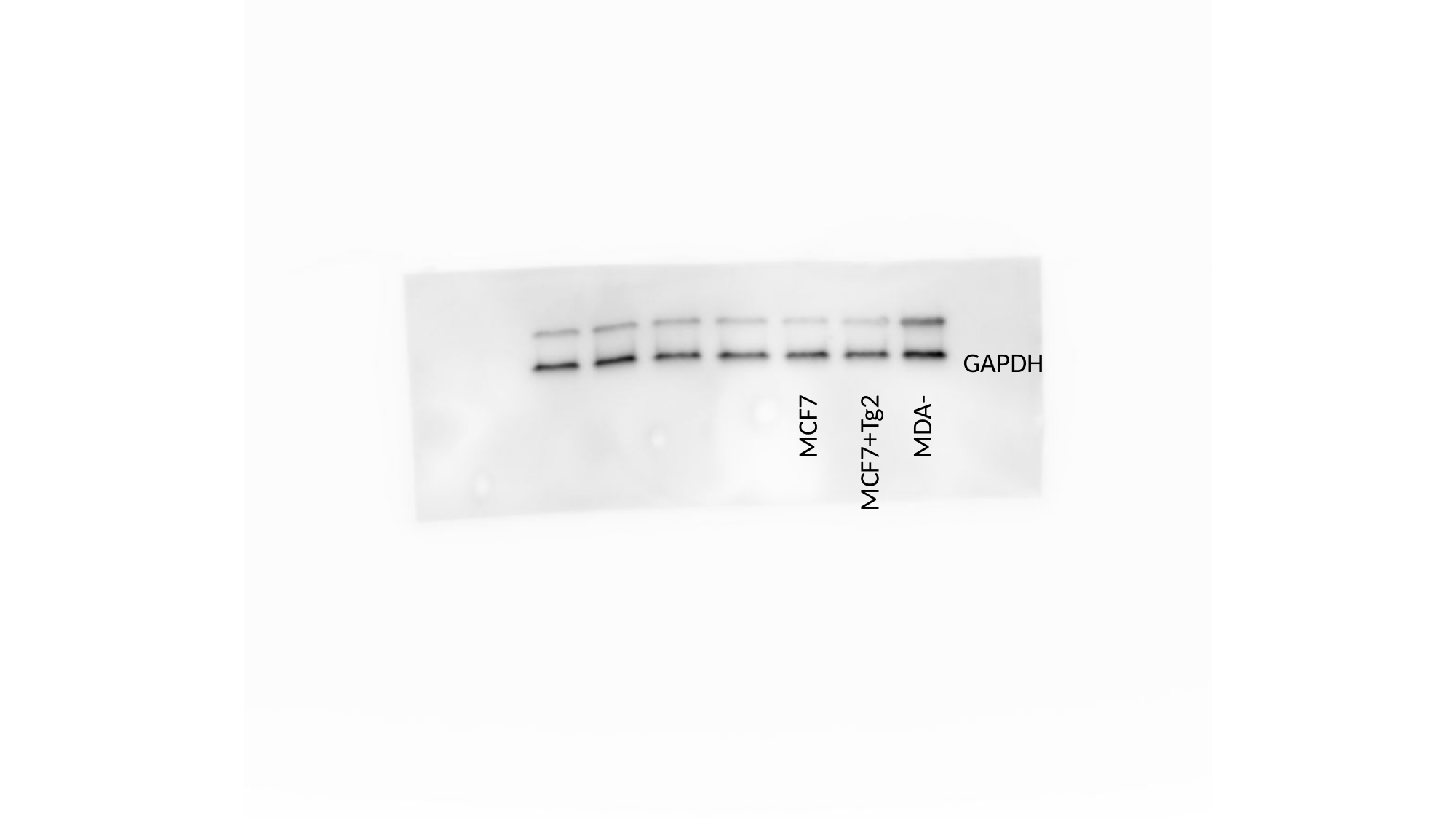

GAPDH
MCF7
MDA-
MCF7+Tg2

## Slide 21
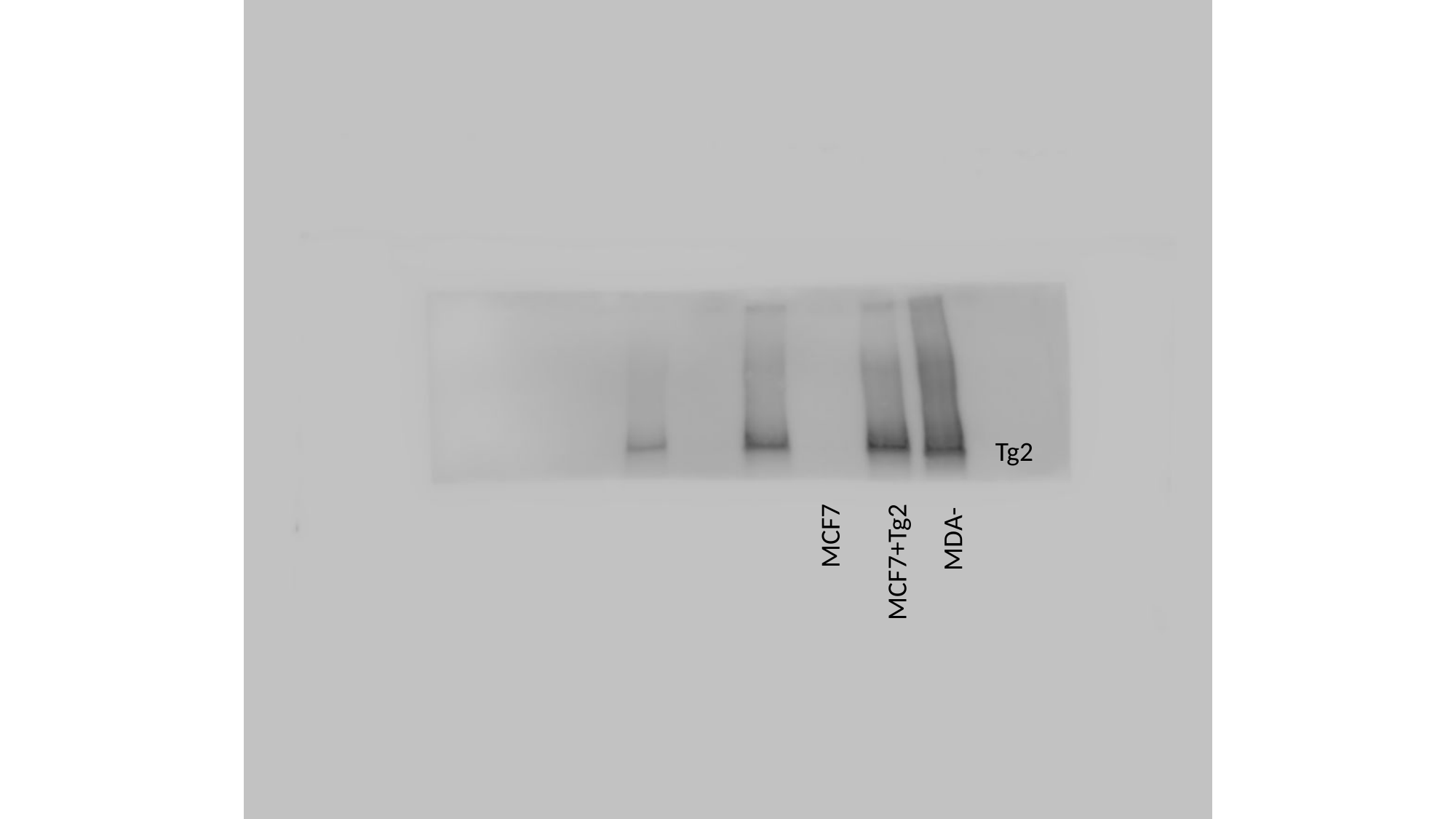

Tg2
MCF7
MDA-
MCF7+Tg2
