# Supplementary figures and images for: Weakly migratory metastatic breast cancer cells activate fibroblasts via microvesicle-Tg2 to facilitate dissemination and metastasis
Source: eLife. 2022 Dec 7;11:e74433. doi: 10.7554/eLife.74433 (PMC9767463; doi:10.7554/eLife.74433)

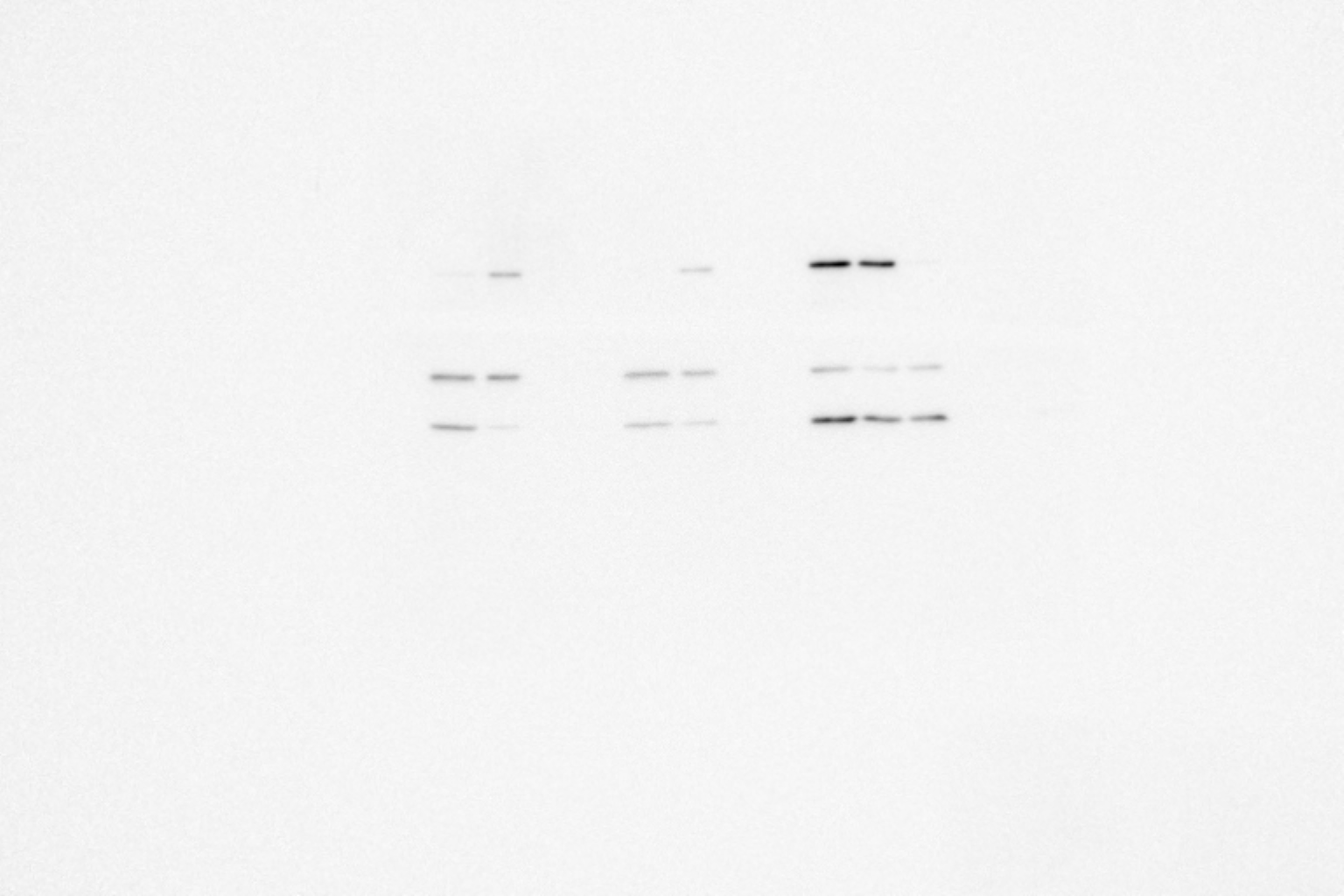

Supplement: Source data 1. [file elife-74433-data1.zip › Western Blots/Figure 4 - SuppFig 1 - A - Western Blots Raw Files/Figure 3 - I - TG2 GAPDH TG2KDINCELLS 011919.tif]

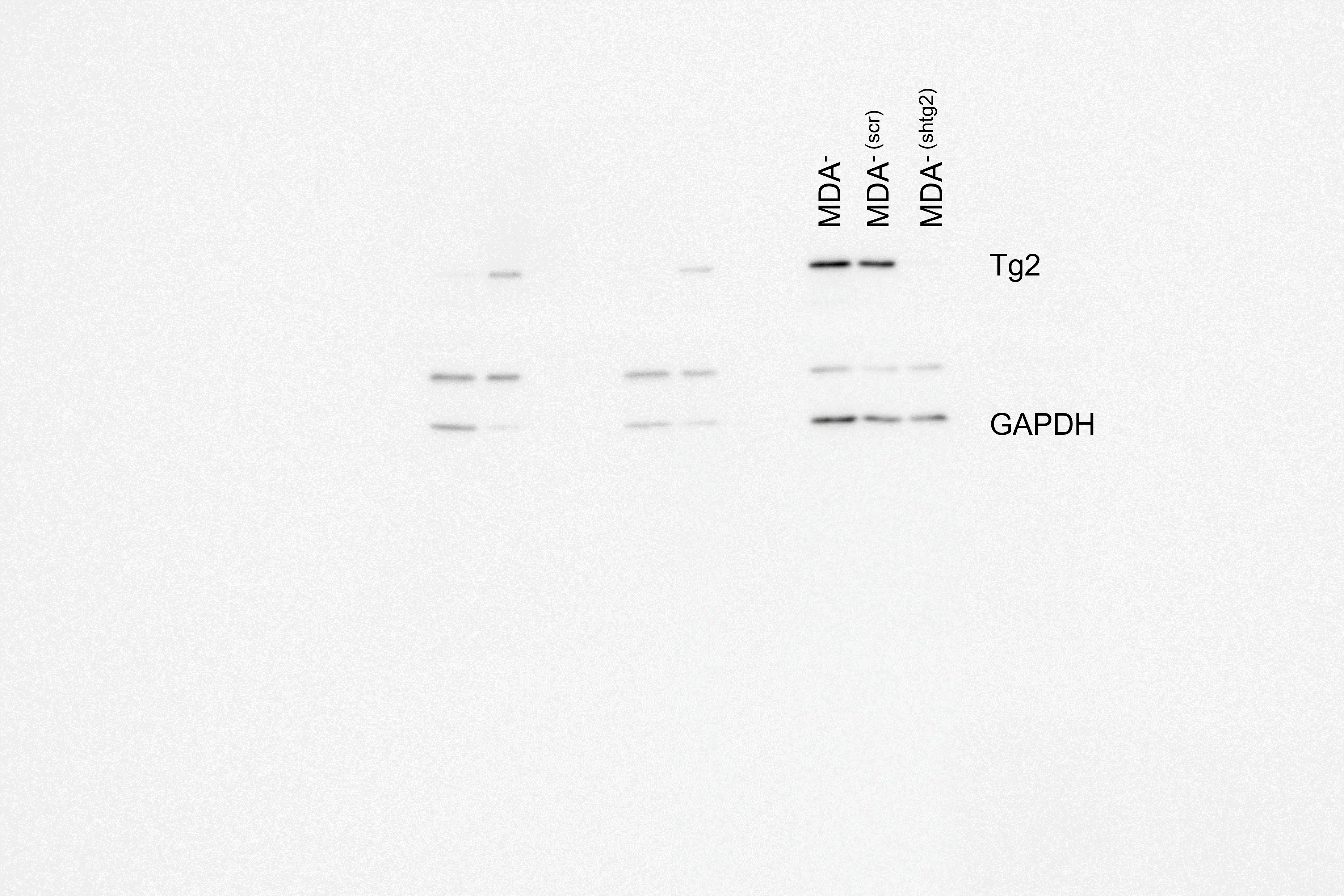

Supplement: Source data 1. [file elife-74433-data1.zip › Western Blots/Figure 4 - SuppFig 1 - A - Western Blots Raw Files/Figure 3 - I Tg2 and GAPDH.png]

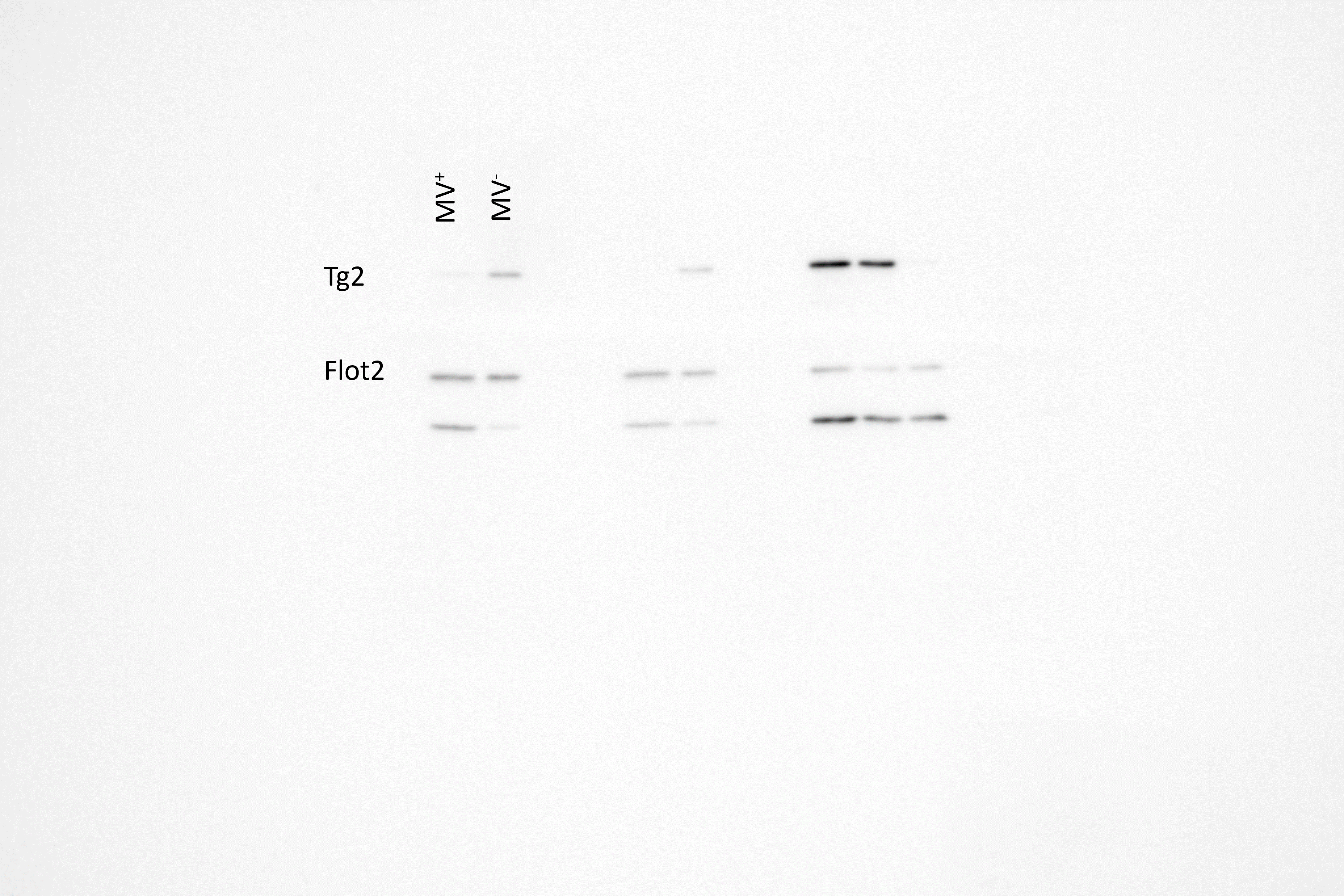

Supplement: Source data 1. [file elife-74433-data1.zip › Western Blots/Figure 3 - G - Western Blots Raw Files/Figure 3 - G Tg2 and Flot2.png]

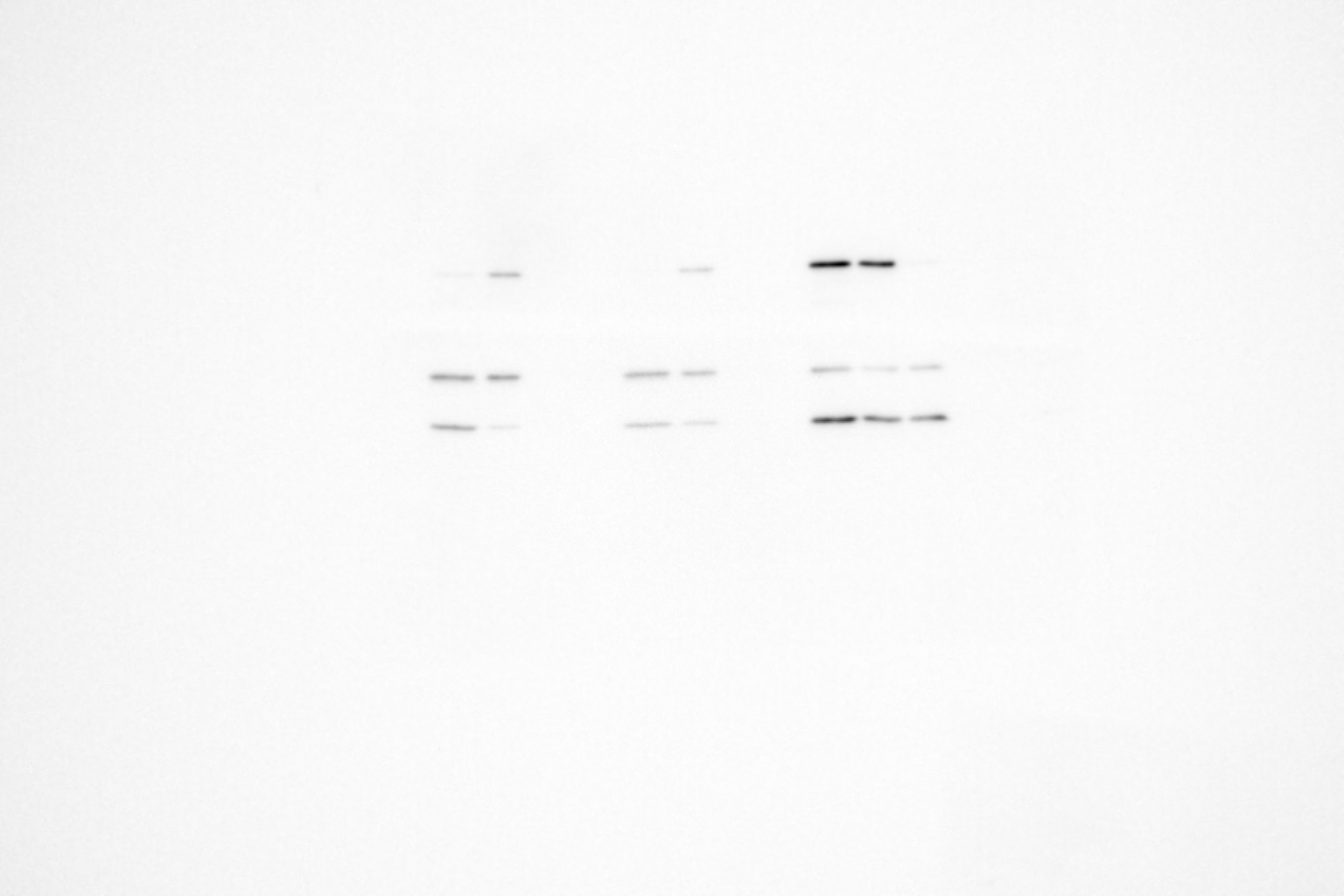

Supplement: Source data 1. [file elife-74433-data1.zip › Western Blots/Figure 3 - G - Western Blots Raw Files/Figure 3 - G - TG2 FLOT2 TG2EXPRESSIONINSUBPOPMVS 011919.tif]

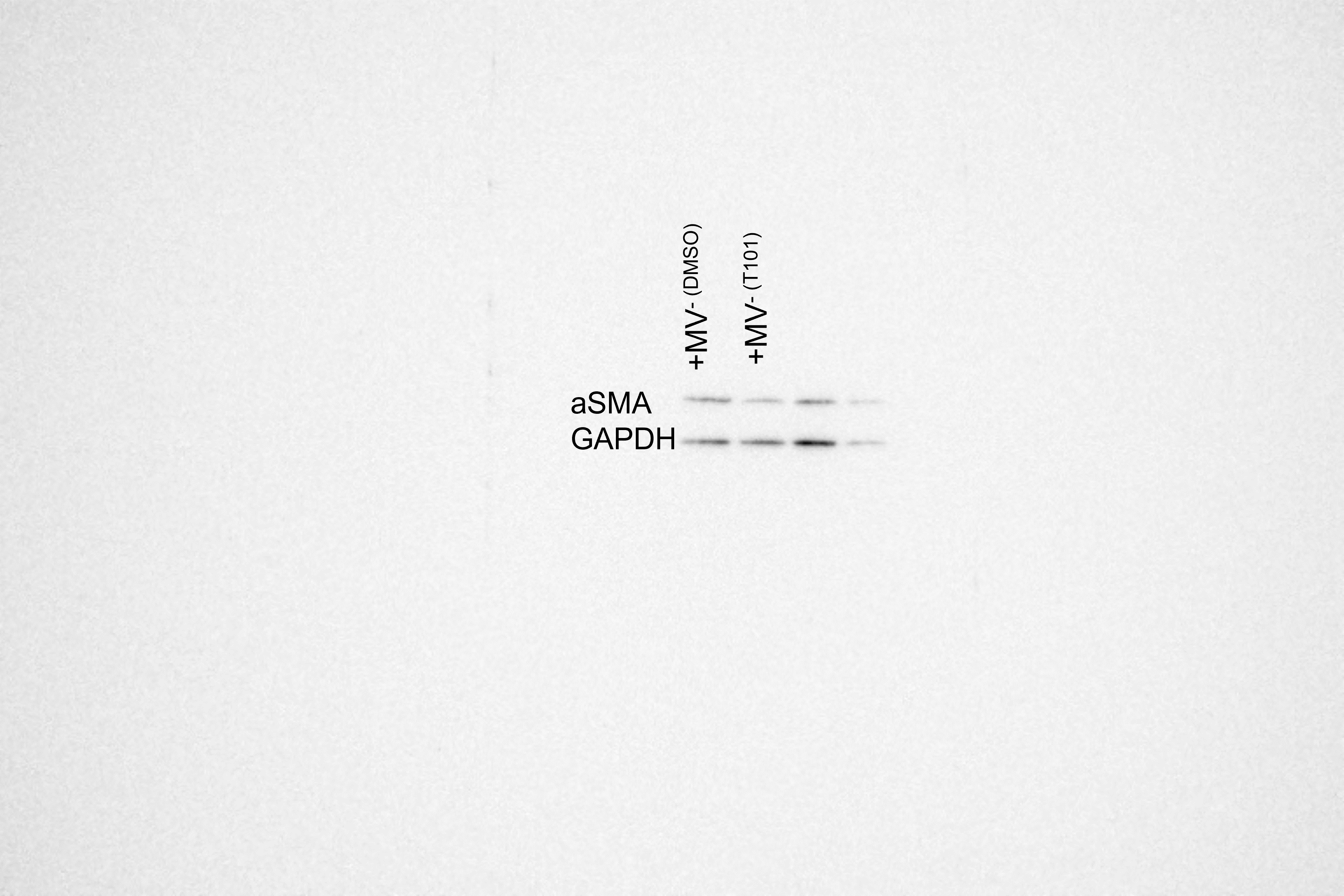

Supplement: Source data 1. [file elife-74433-data1.zip › Western Blots/Figure 4 - H - Western Blots Raw Files/Figure 4 - H - aSMA and GAPDH.png]

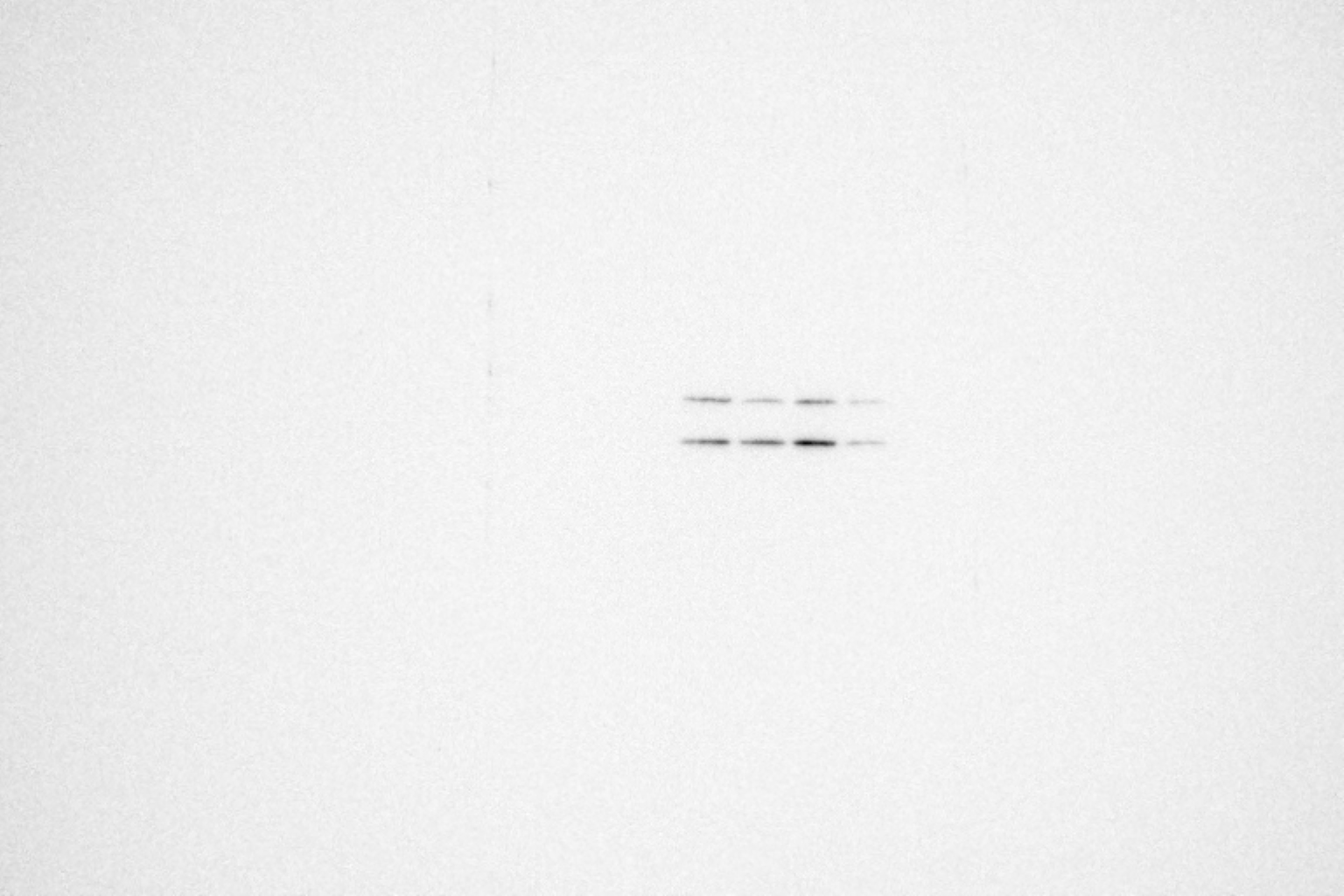

Supplement: Source data 1. [file elife-74433-data1.zip › Western Blots/Figure 4 - H - Western Blots Raw Files/Figure 4 - H - ASMA GAPDH T101INHIBITOR 071719.tif]

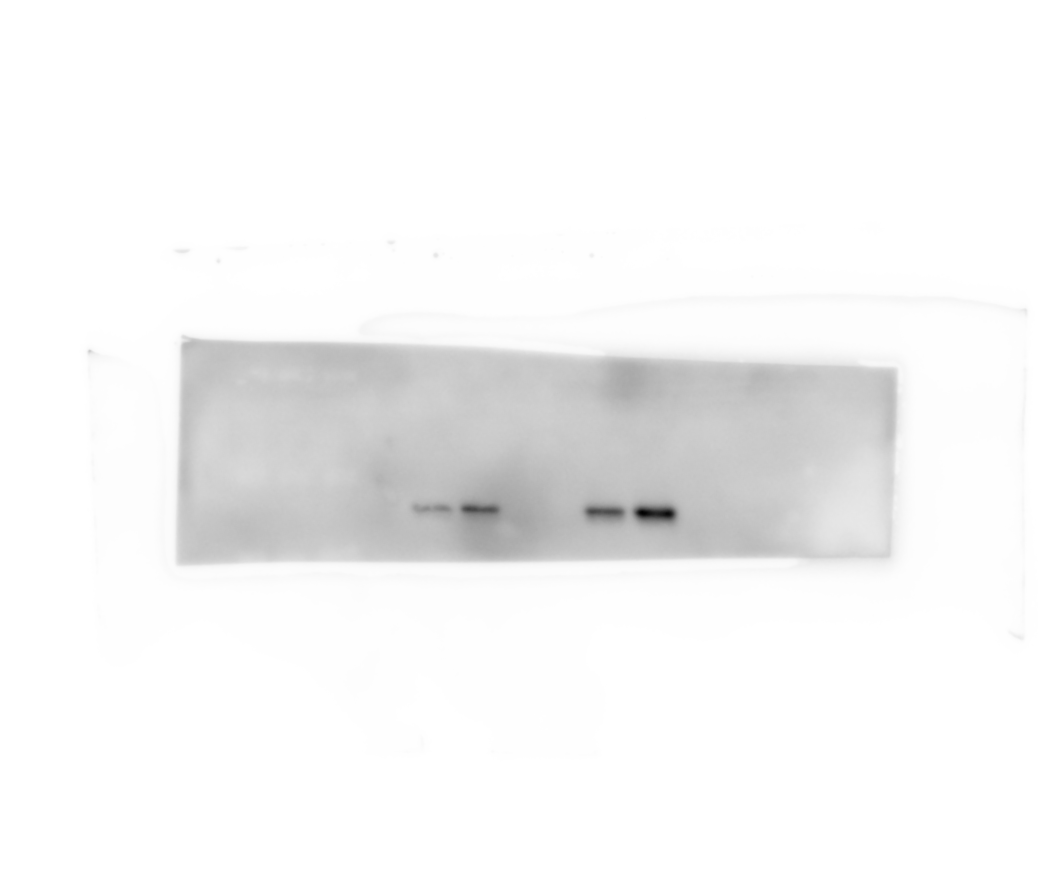

Supplement: Source data 1. [file elife-74433-data1.zip › Western Blots/Figure 3 - SuppFig 1 - E - Western Blot Raw Files/Supp Figure 2 - E - FLOT2 FNINSUBPOPMVS 050821.tif]

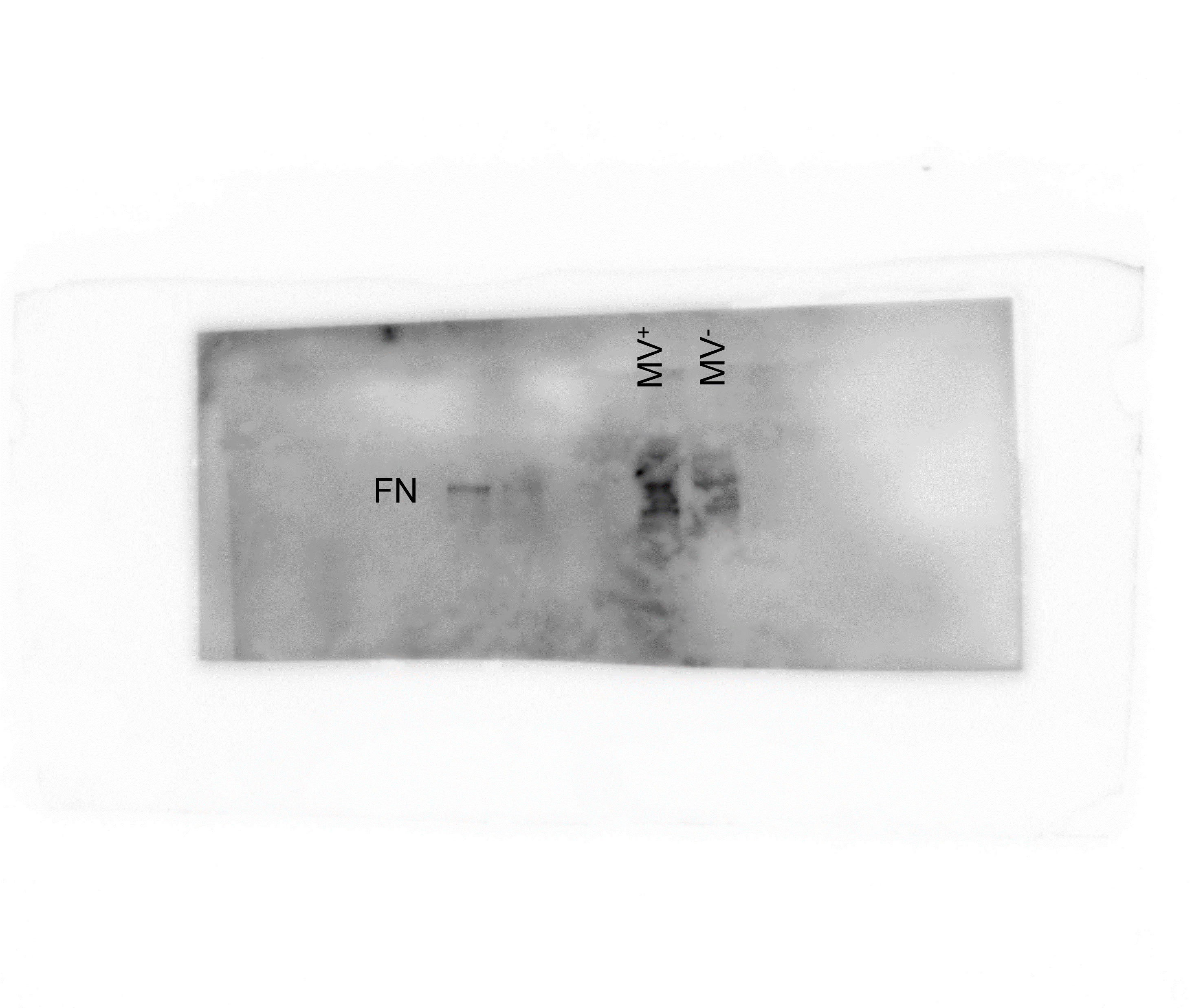

Supplement: Source data 1. [file elife-74433-data1.zip › Western Blots/Figure 3 - SuppFig 1 - E - Western Blot Raw Files/Supp Figure 2 - E - FN.png]

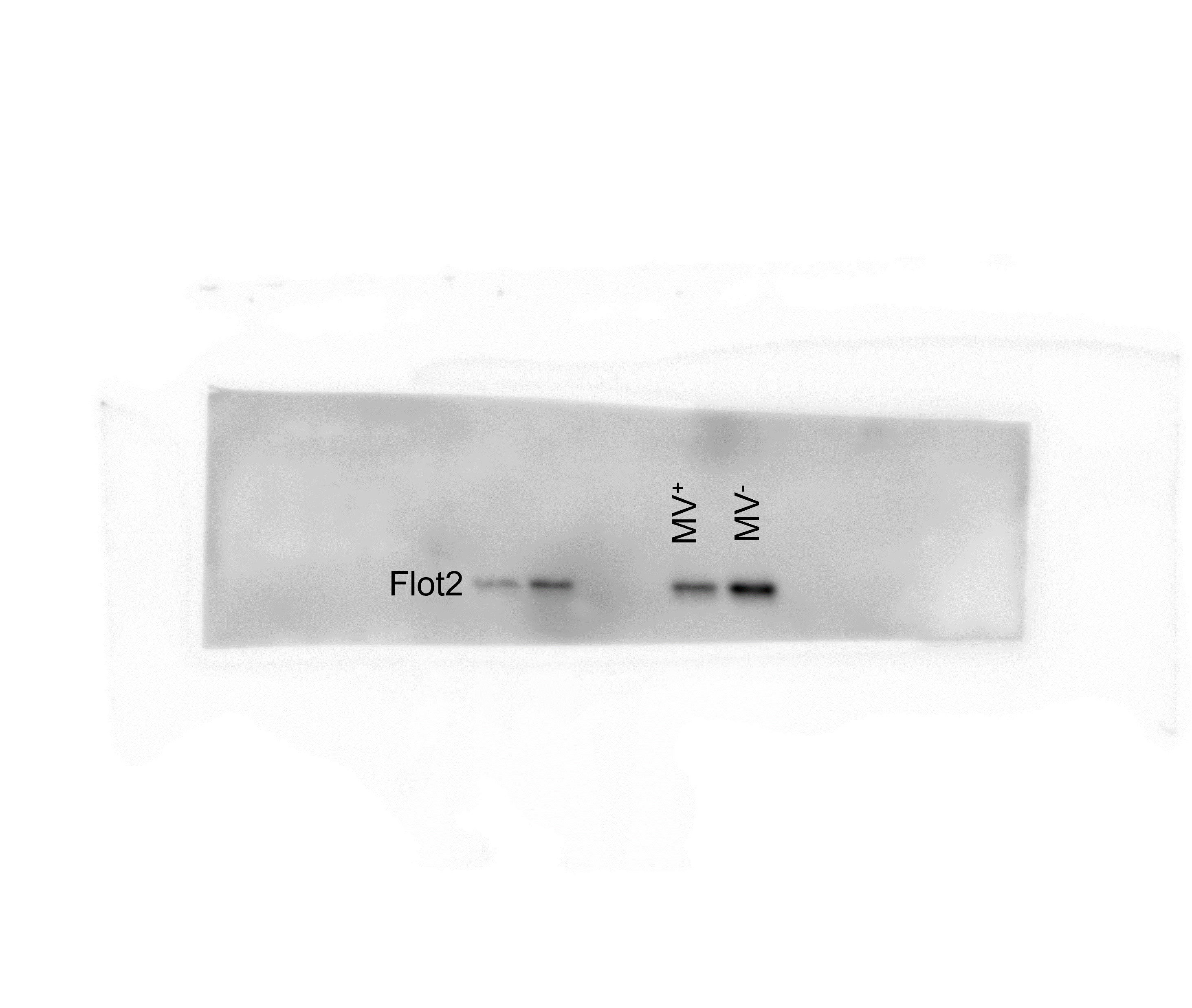

Supplement: Source data 1. [file elife-74433-data1.zip › Western Blots/Figure 3 - SuppFig 1 - E - Western Blot Raw Files/Supp Figure 2 - E - Flot2.png]

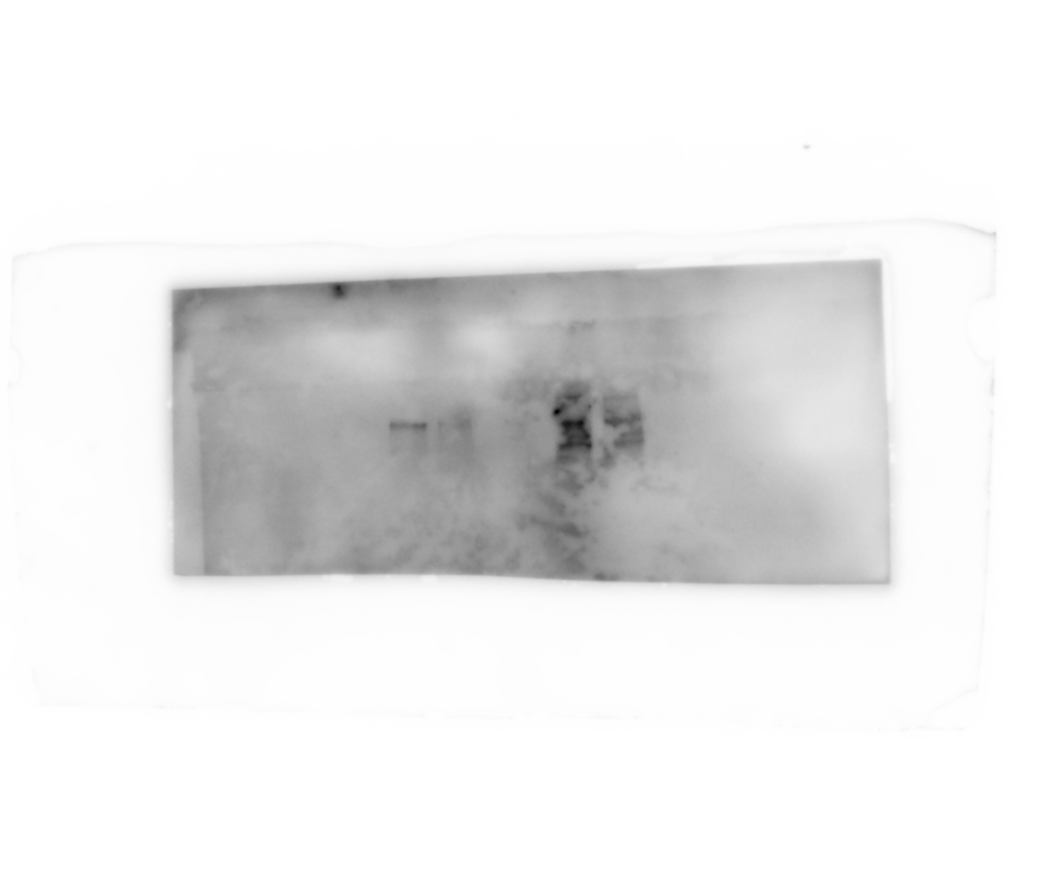

Supplement: Source data 1. [file elife-74433-data1.zip › Western Blots/Figure 3 - SuppFig 1 - E - Western Blot Raw Files/Supp Figure 2 - E - FN FNINSUBPOPMVS 050821.tif]

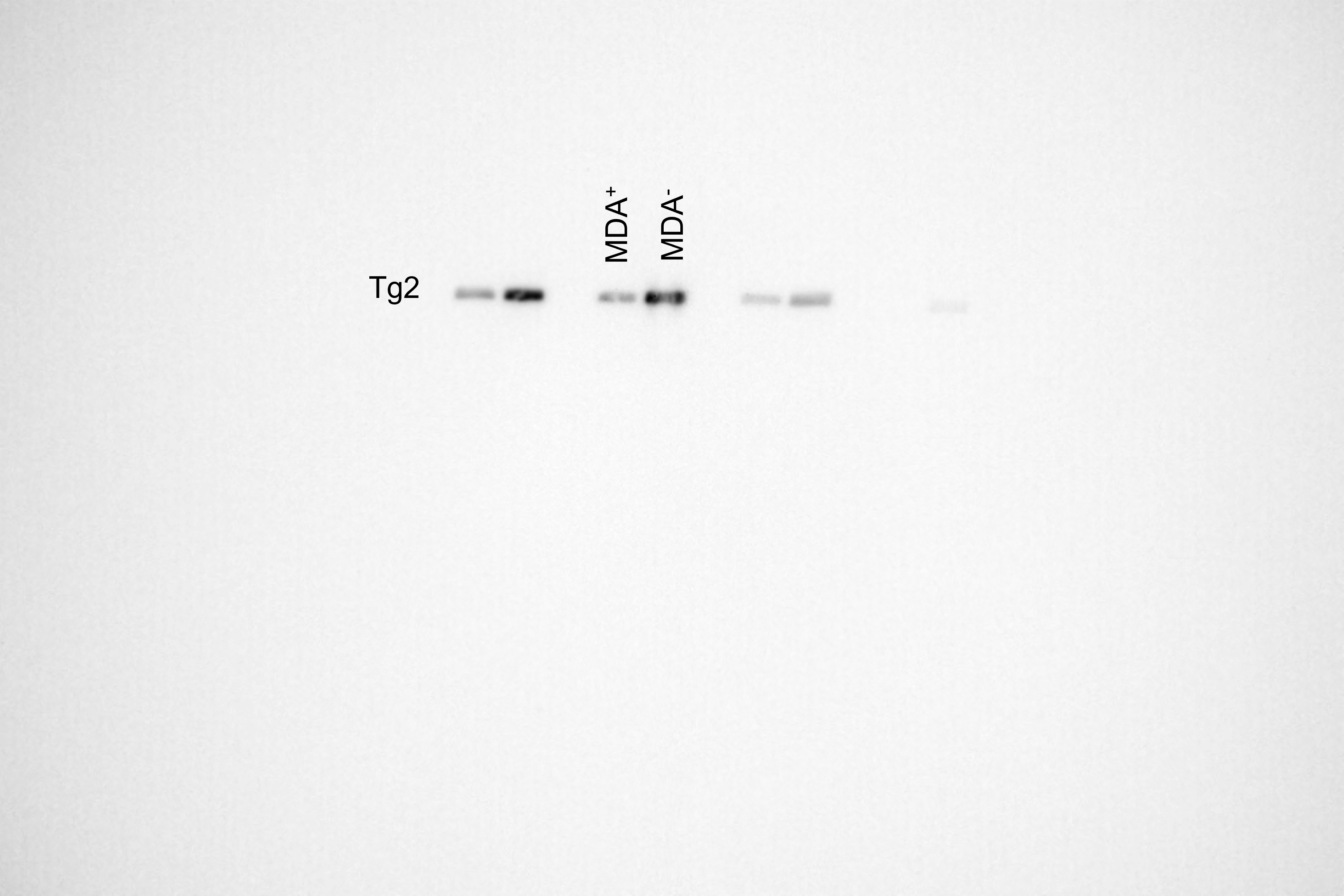

Supplement: Source data 1. [file elife-74433-data1.zip › Western Blots/Figure 3 - E - Western Blots Raw Files/Figure 3 - E Tg2.png]

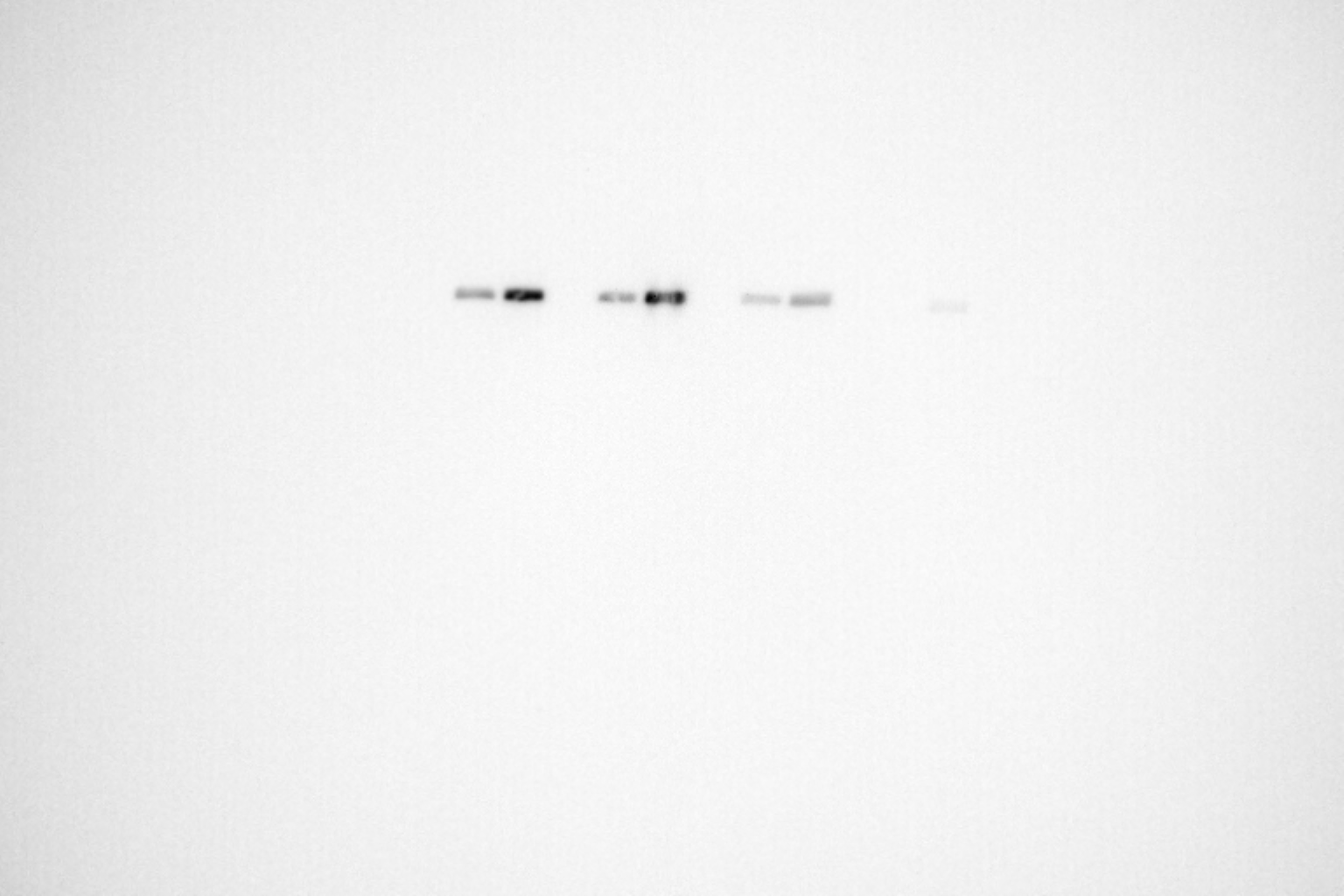

Supplement: Source data 1. [file elife-74433-data1.zip › Western Blots/Figure 3 - E - Western Blots Raw Files/Figure 3 - E - TG2 TG2INSUBPOPS 042319.tif]

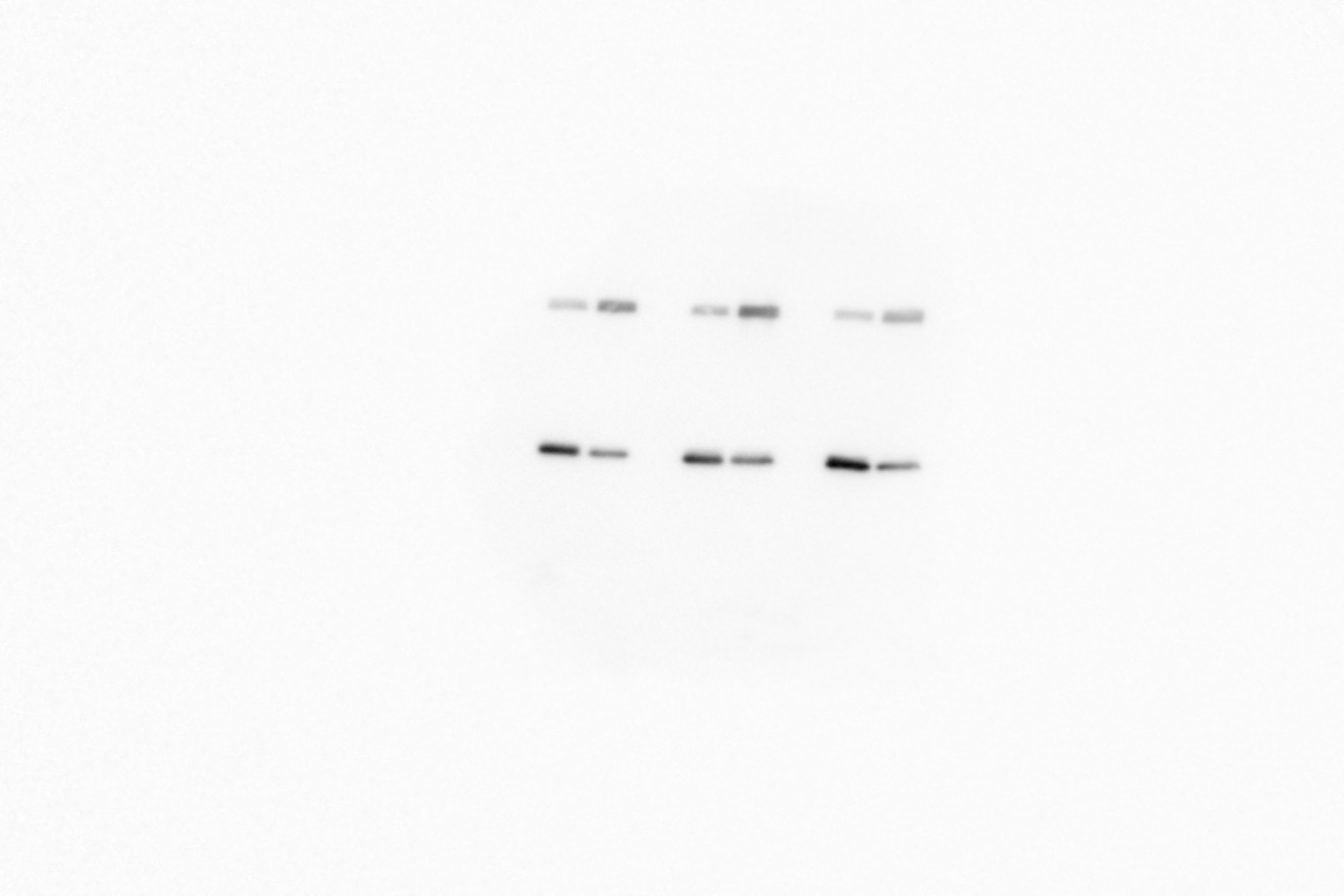

Supplement: Source data 1. [file elife-74433-data1.zip › Western Blots/Figure 3 - E - Western Blots Raw Files/Figure 3 - E - GAPDH TG2INSUBPOPS 042419.tif]

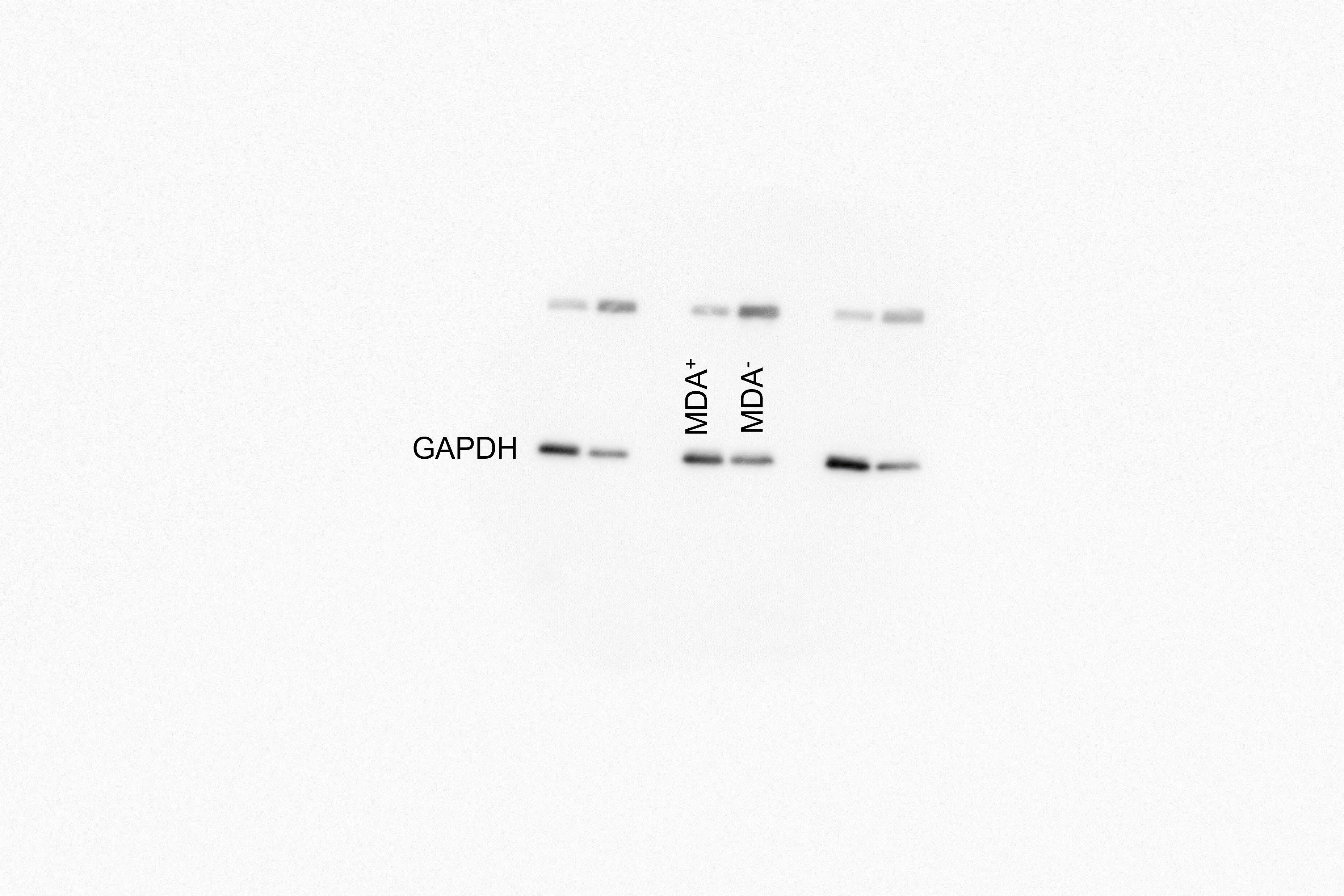

Supplement: Source data 1. [file elife-74433-data1.zip › Western Blots/Figure 3 - E - Western Blots Raw Files/Figure 3 - E GAPDH.png]

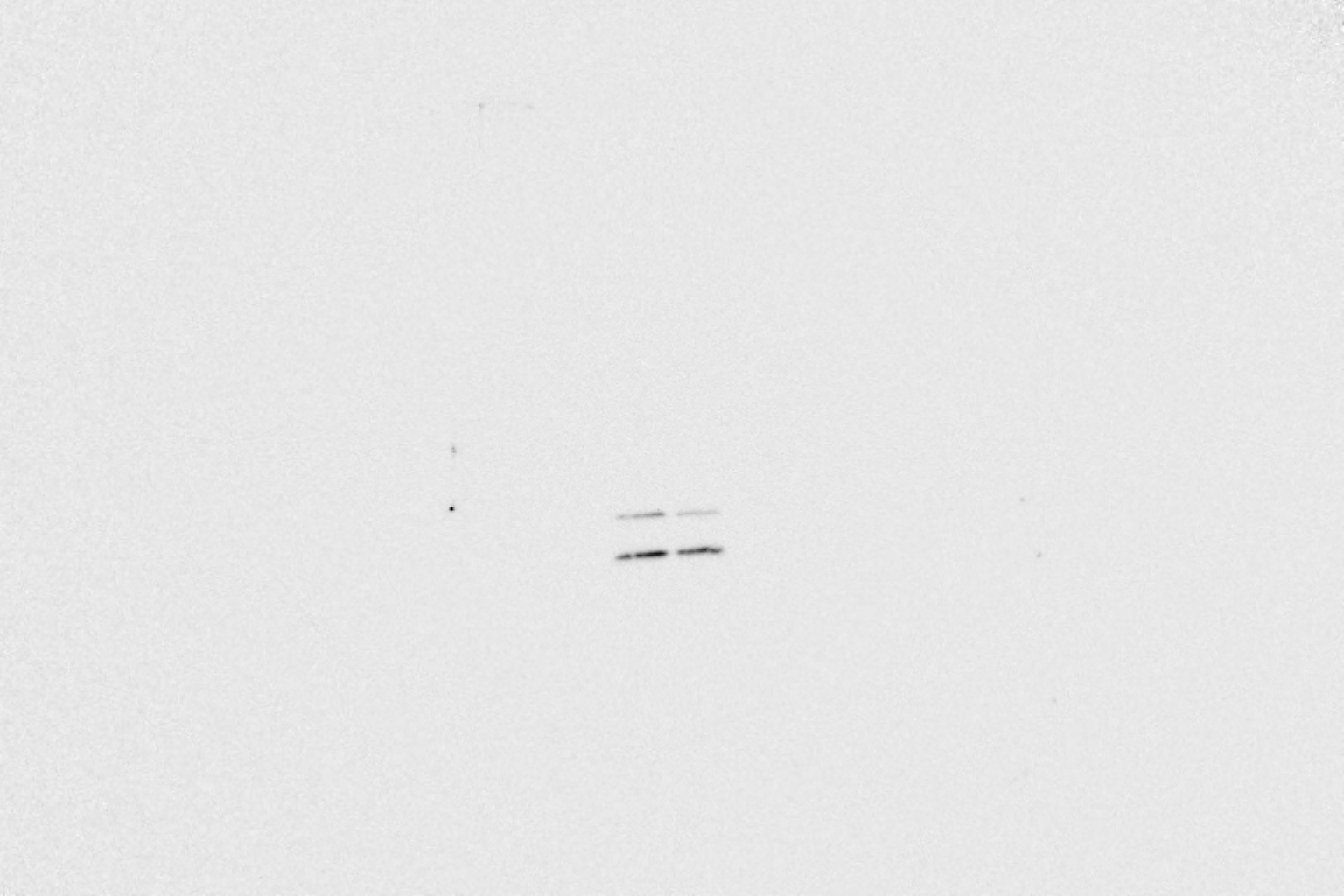

Supplement: Source data 1. [file elife-74433-data1.zip › Western Blots/Figure 4 - B - Western Blots Raw Files/Figure 4 - B - ASMA GAPDH 3T3WITHSHTG2MV 071719.tif]

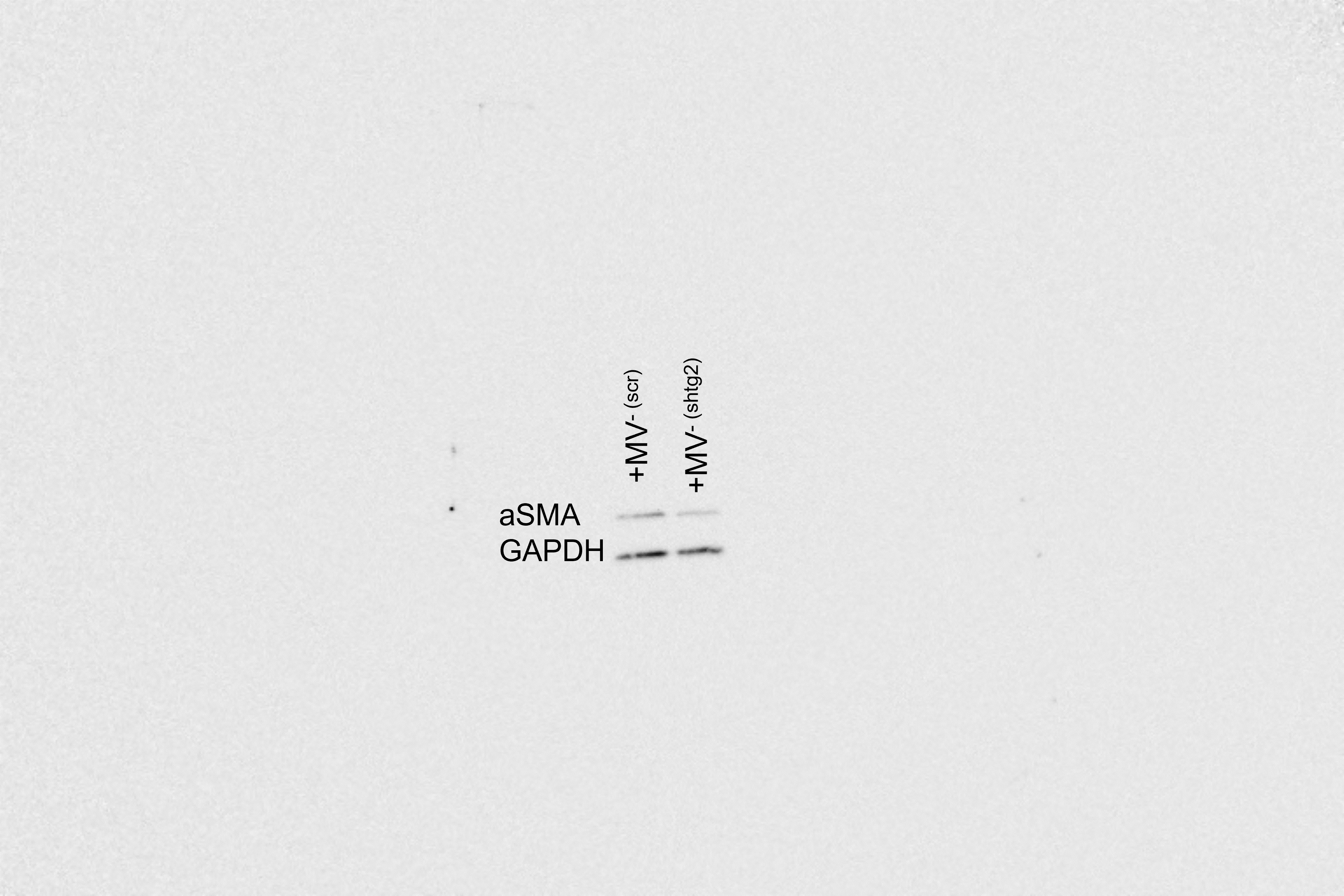

Supplement: Source data 1. [file elife-74433-data1.zip › Western Blots/Figure 4 - B - Western Blots Raw Files/Figure 4 - B aSMA and GAPDH.png]

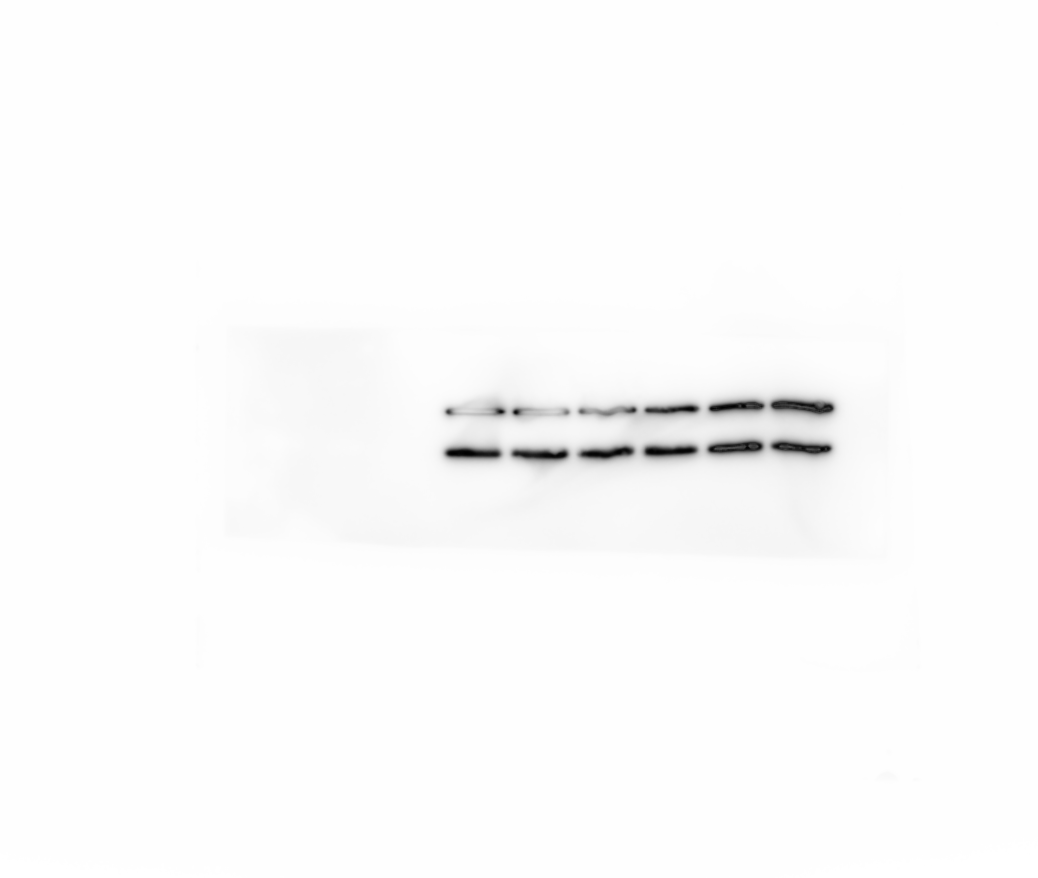

Supplement: Source data 1. [file elife-74433-data1.zip › Western Blots/Figure 2 - F - Western Blots Raw Files/Figure 2 - F - aSMA GAPDH - new.tif]

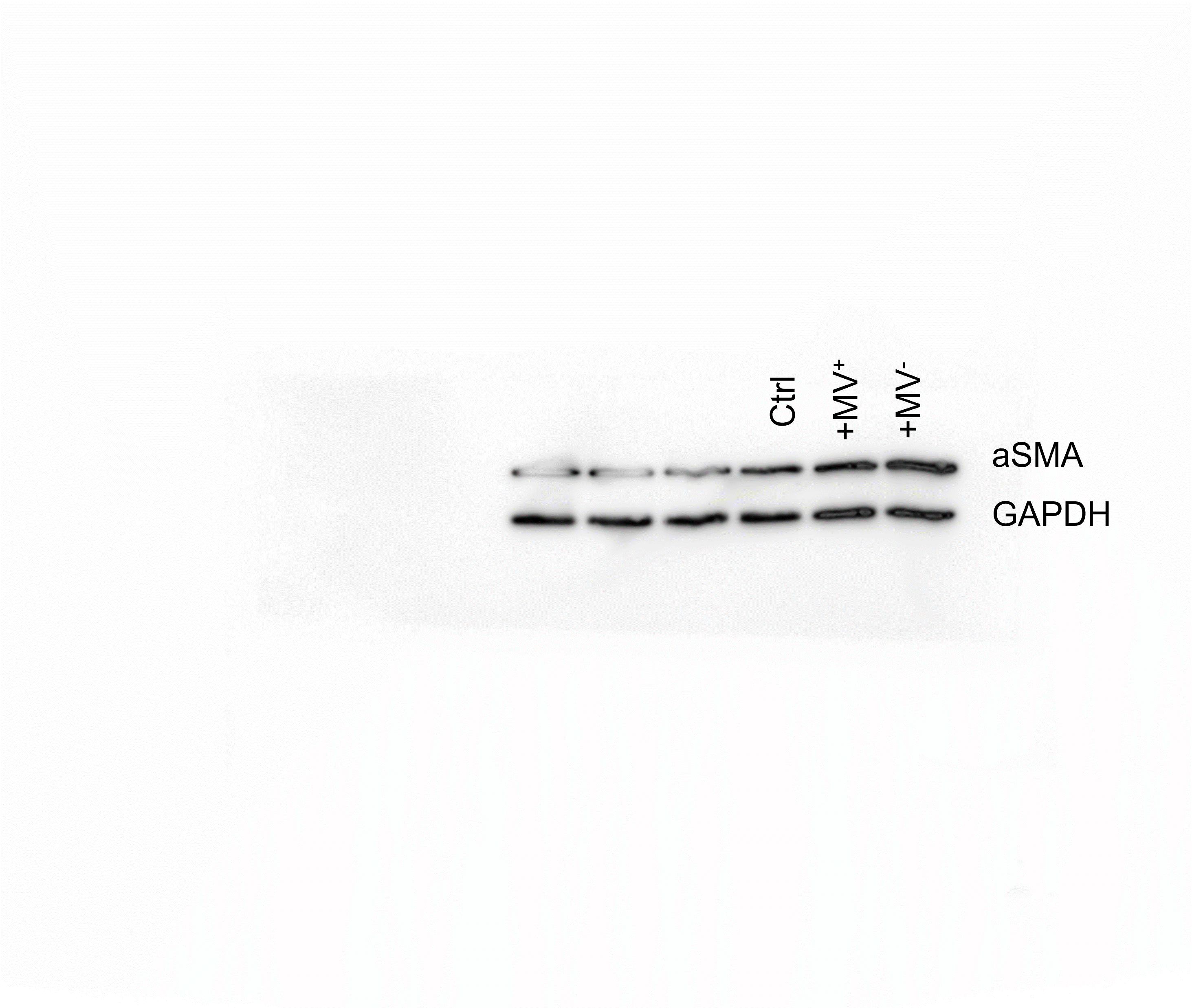

Supplement: Source data 1. [file elife-74433-data1.zip › Western Blots/Figure 2 - F - Western Blots Raw Files/Figure 2 - F - aSMA GAPDH - new with labels.tif.png]

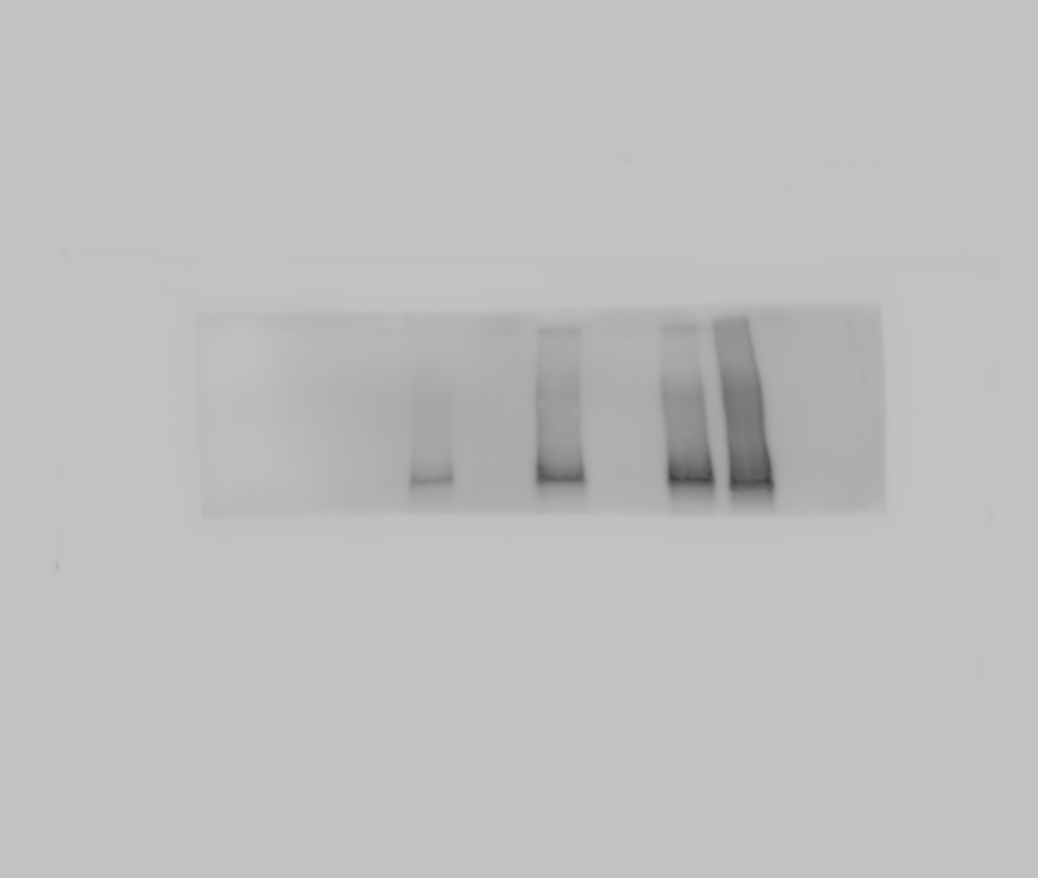

Supplement: Source data 1. [file elife-74433-data1.zip › Western Blots/Figure 4 - SuppFig 3 - D - Western Blot Raw Files/Supp Fig 5 - D - Tg2.tif]

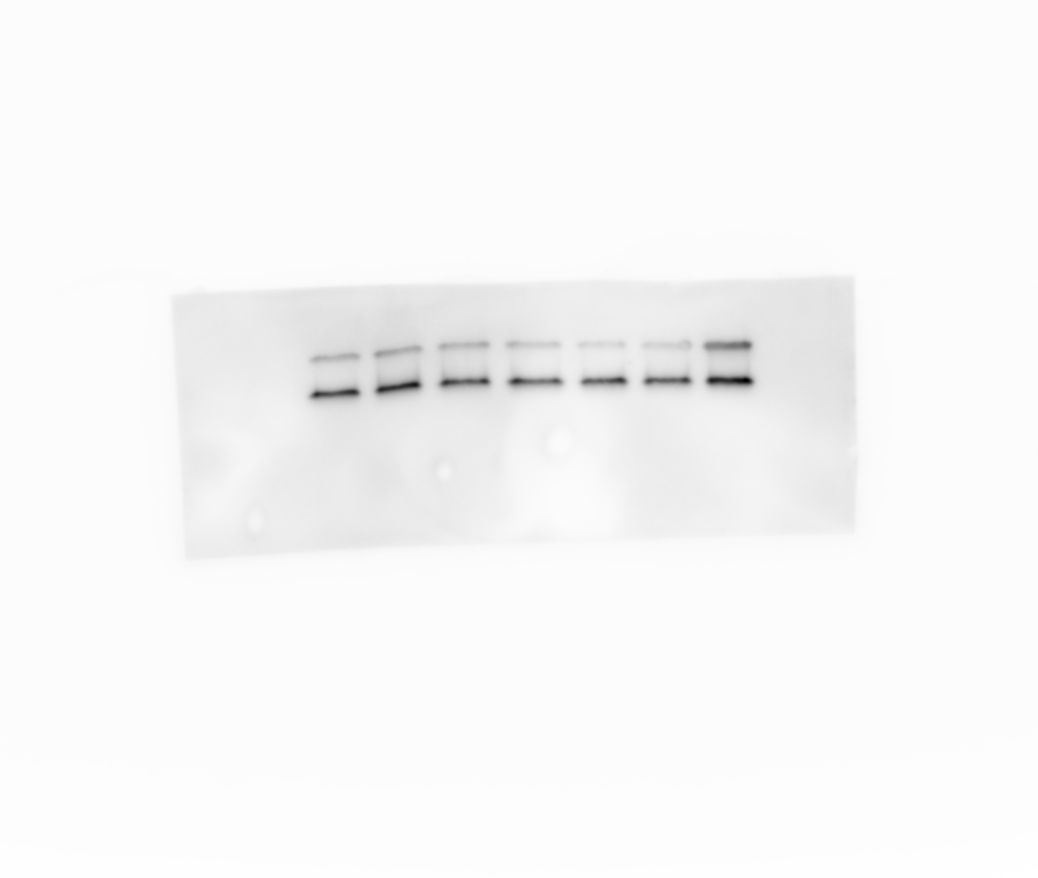

Supplement: Source data 1. [file elife-74433-data1.zip › Western Blots/Figure 4 - SuppFig 3 - D - Western Blot Raw Files/Supp Fig 5 - D - GAPDH.tif]

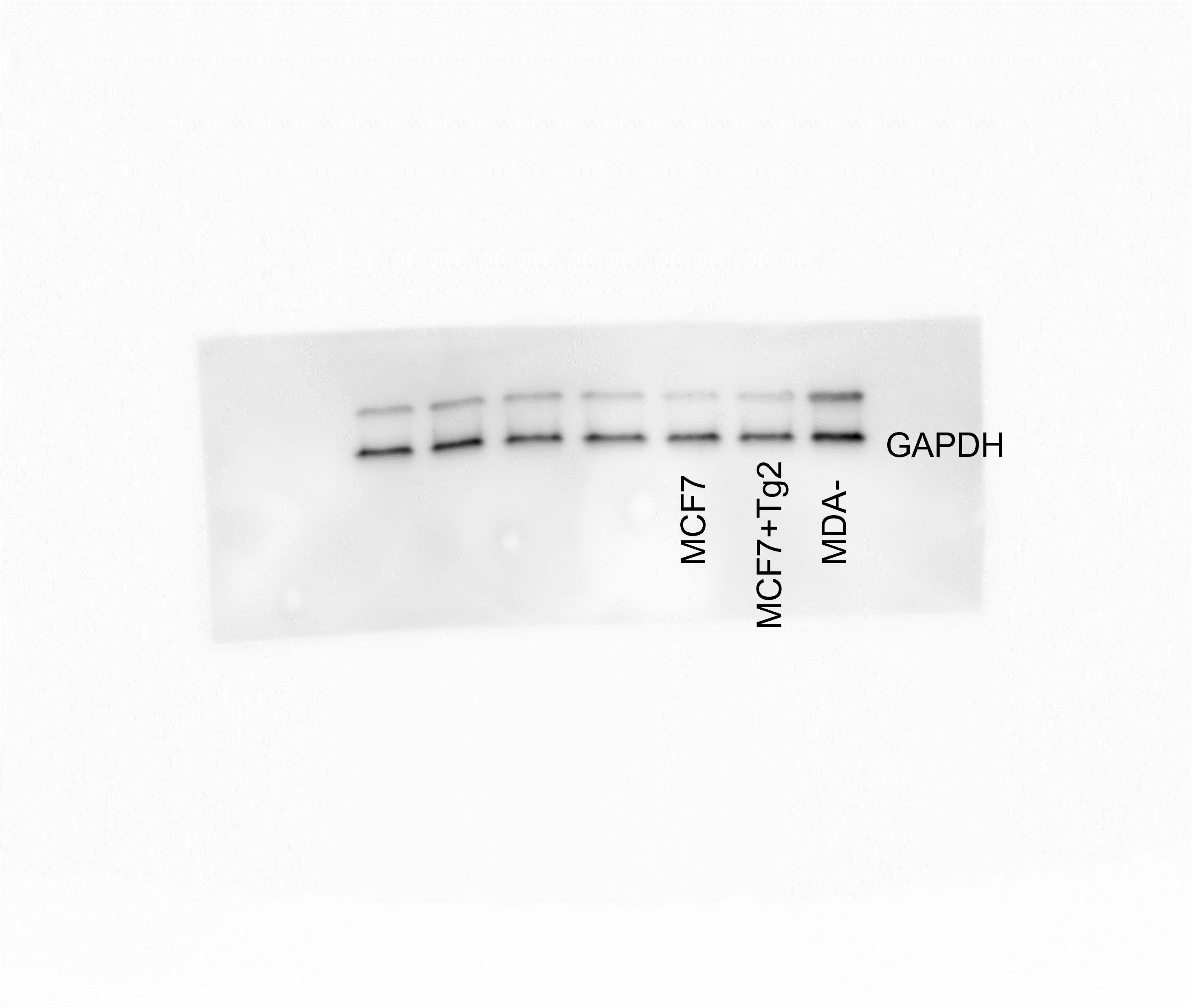

Supplement: Source data 1. [file elife-74433-data1.zip › Western Blots/Figure 4 - SuppFig 3 - D - Western Blot Raw Files/Supp Fig 5 - D - GAPDH - labels.tif.png]

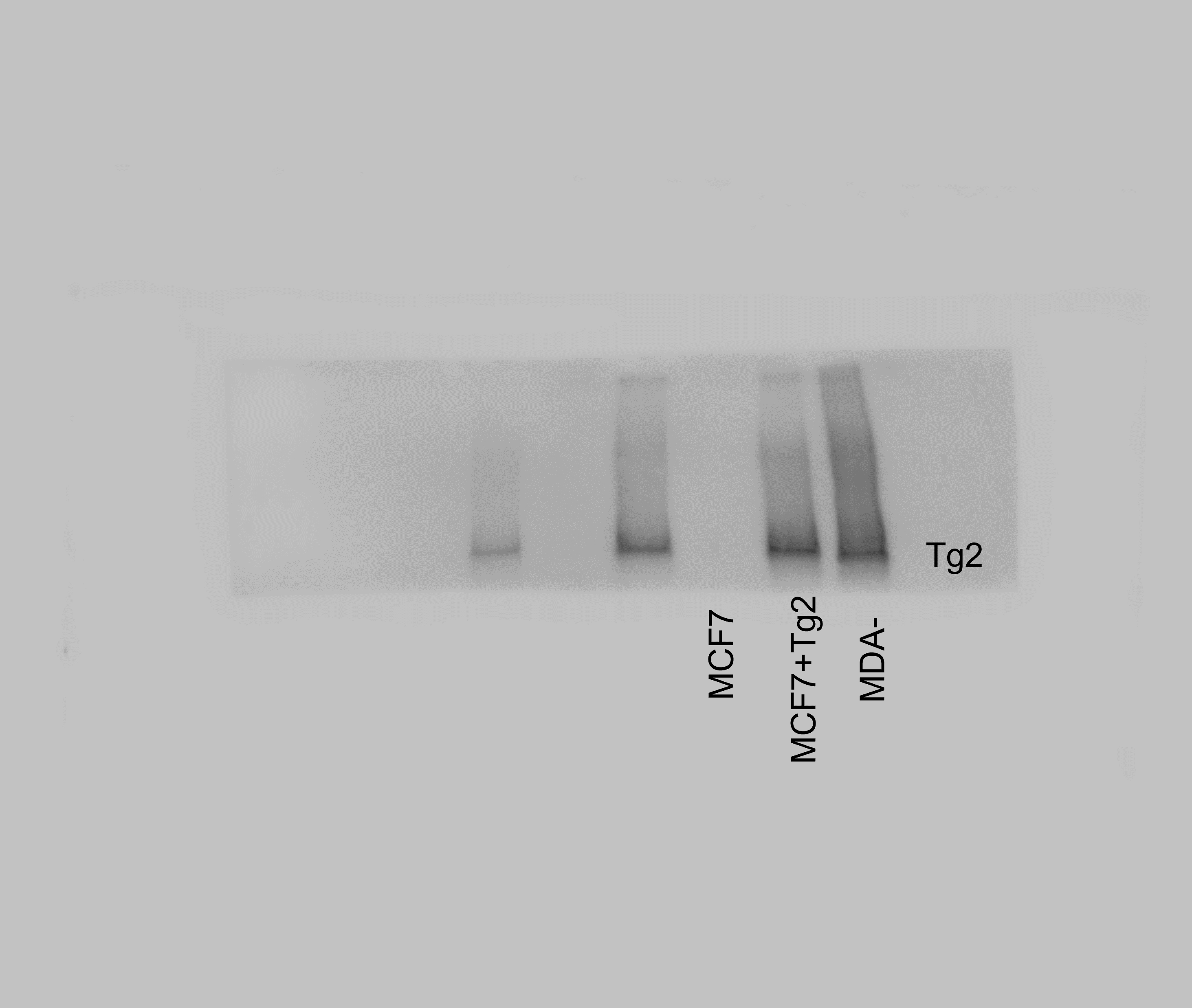

Supplement: Source data 1. [file elife-74433-data1.zip › Western Blots/Figure 4 - SuppFig 3 - D - Western Blot Raw Files/Supp Fig 5 - D - Tg2 - labels.tif.png]

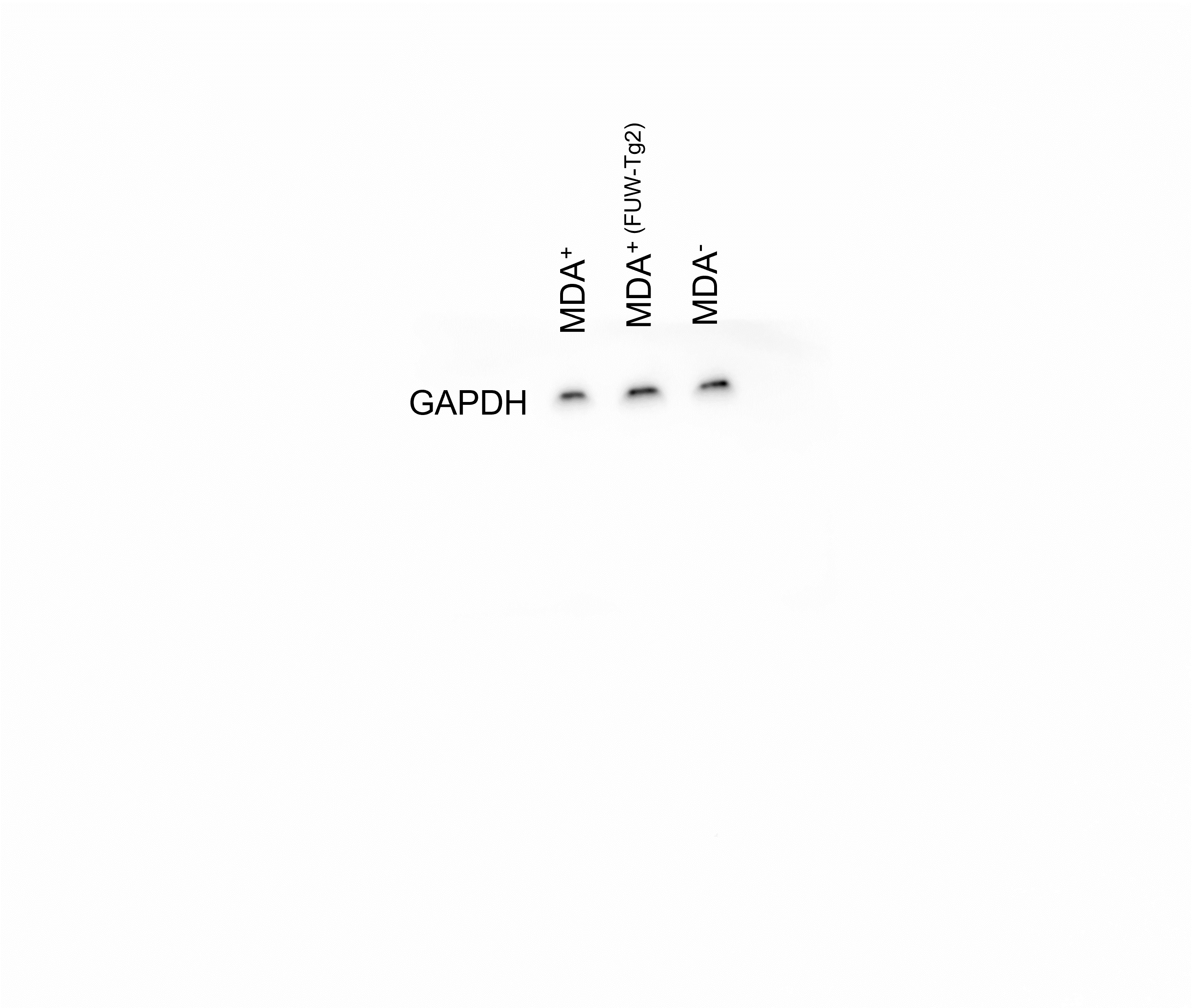

Supplement: Source data 1. [file elife-74433-data1.zip › Western Blots/Figure 4 - SuppFig 3 - A - Western Blots Raw Files/SuppFig2 - G gapdh.png]

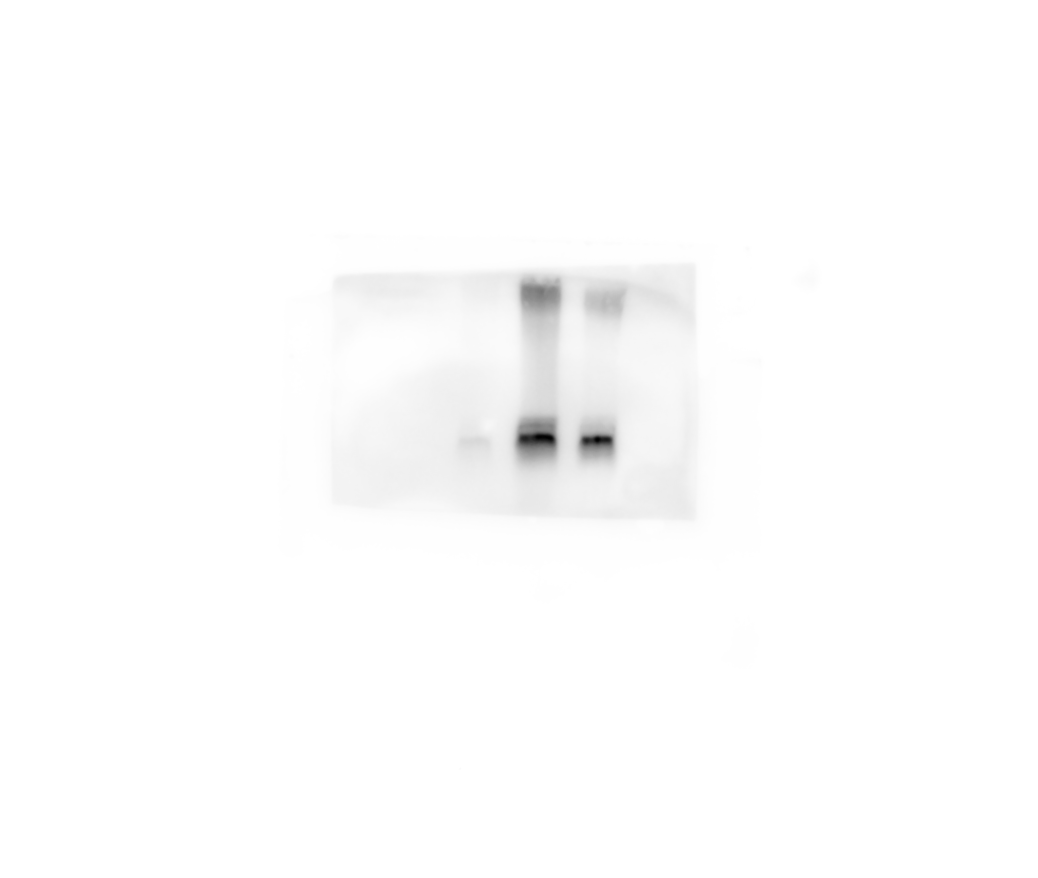

Supplement: Source data 1. [file elife-74433-data1.zip › Western Blots/Figure 4 - SuppFig 3 - A - Western Blots Raw Files/SuppFig 2 - G - TG2 TG2INMDAINV 032821.tif]

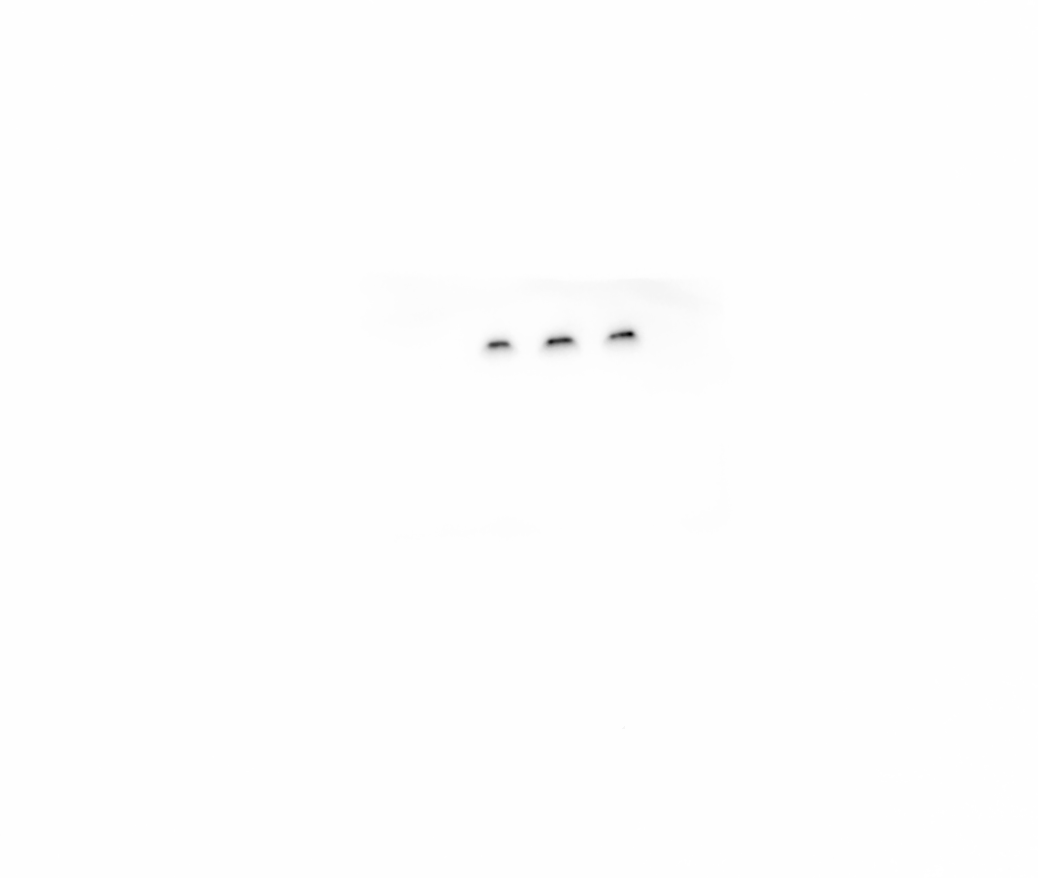

Supplement: Source data 1. [file elife-74433-data1.zip › Western Blots/Figure 4 - SuppFig 3 - A - Western Blots Raw Files/SuppFig 2 - G - GAPDH TG2INMDAINV 032821.tif]

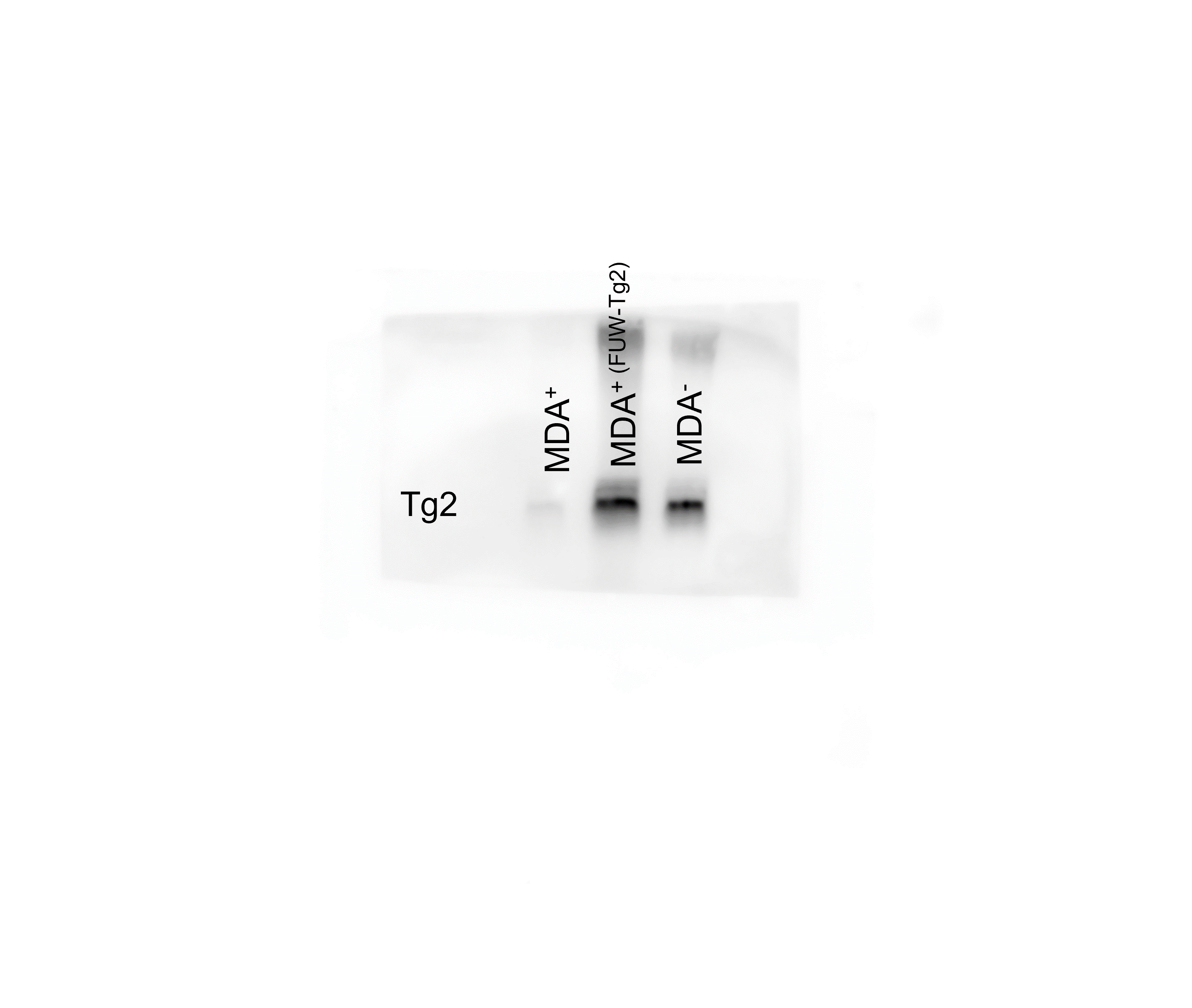

Supplement: Source data 1. [file elife-74433-data1.zip › Western Blots/Figure 4 - SuppFig 3 - A - Western Blots Raw Files/SuppFig2 - G Tg2.png]

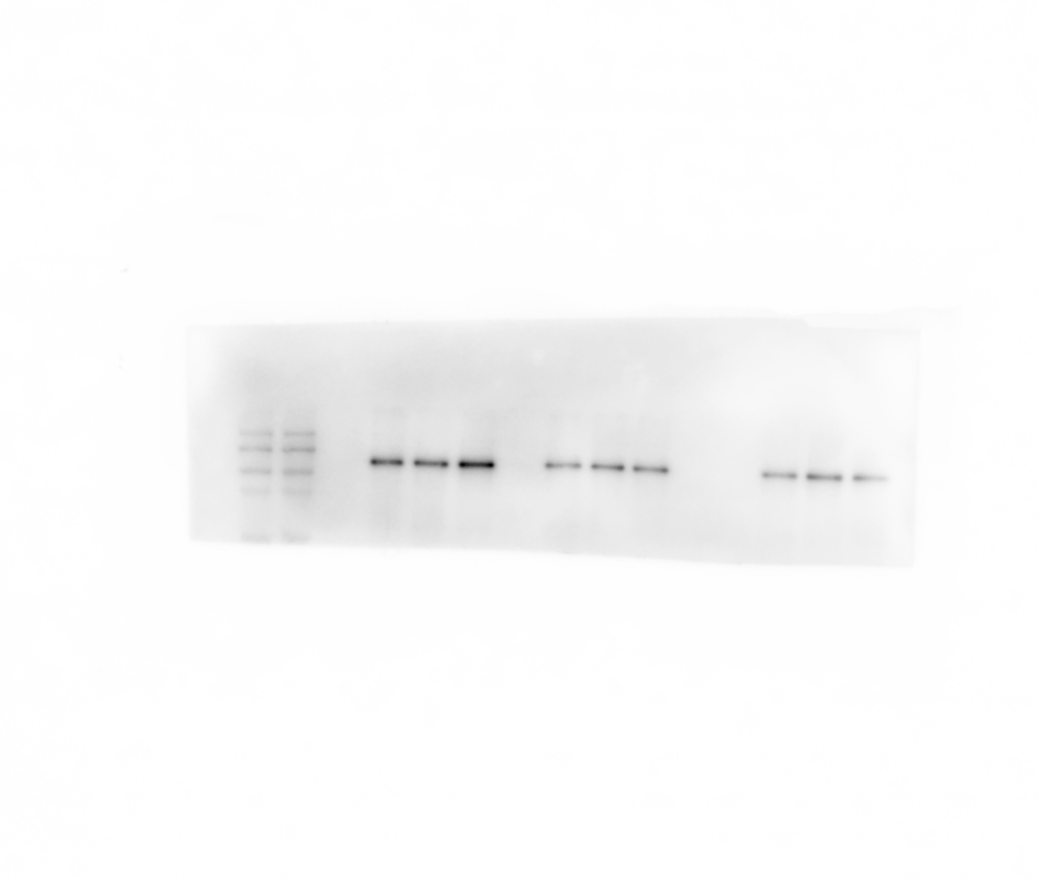

Supplement: Source data 1. [file elife-74433-data1.zip › Western Blots/Figure 2 - D - Western Blots Raw Files/Figure 2 - D - FAK 3T3WITHMVS 052621.tif]

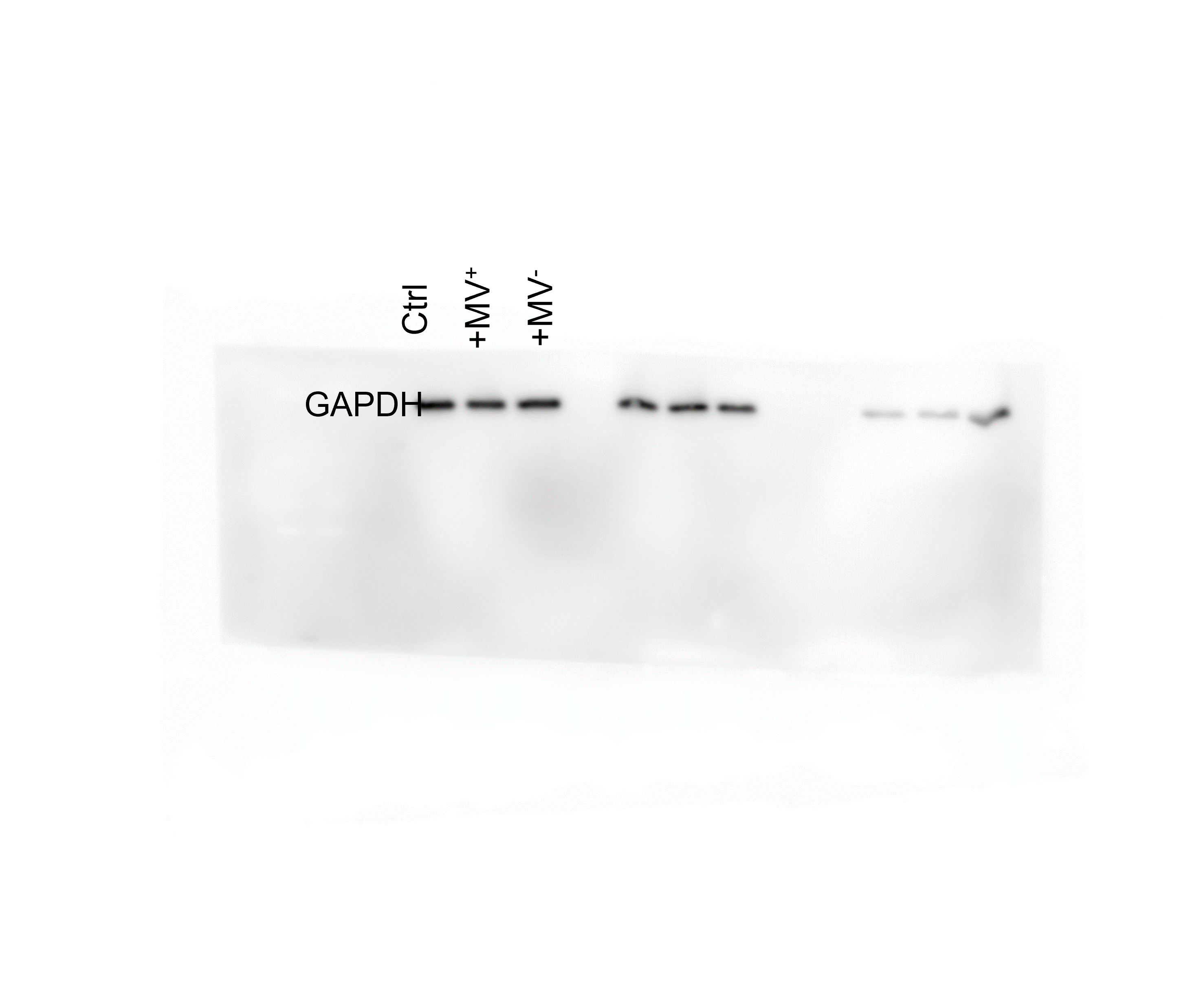

Supplement: Source data 1. [file elife-74433-data1.zip › Western Blots/Figure 2 - D - Western Blots Raw Files/Figure 2 - D GAPDH.png]

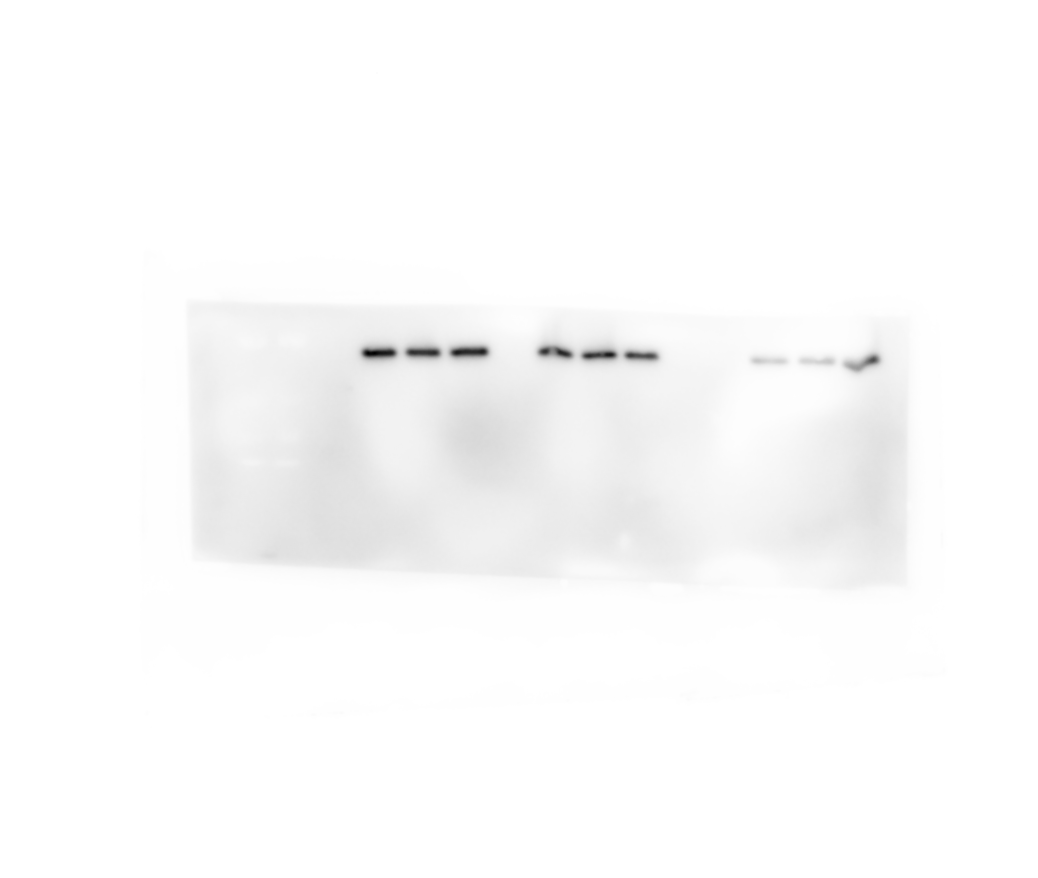

Supplement: Source data 1. [file elife-74433-data1.zip › Western Blots/Figure 2 - D - Western Blots Raw Files/Figure 2 - D - GAPDH 3T3WITHMVS 052621.tif]

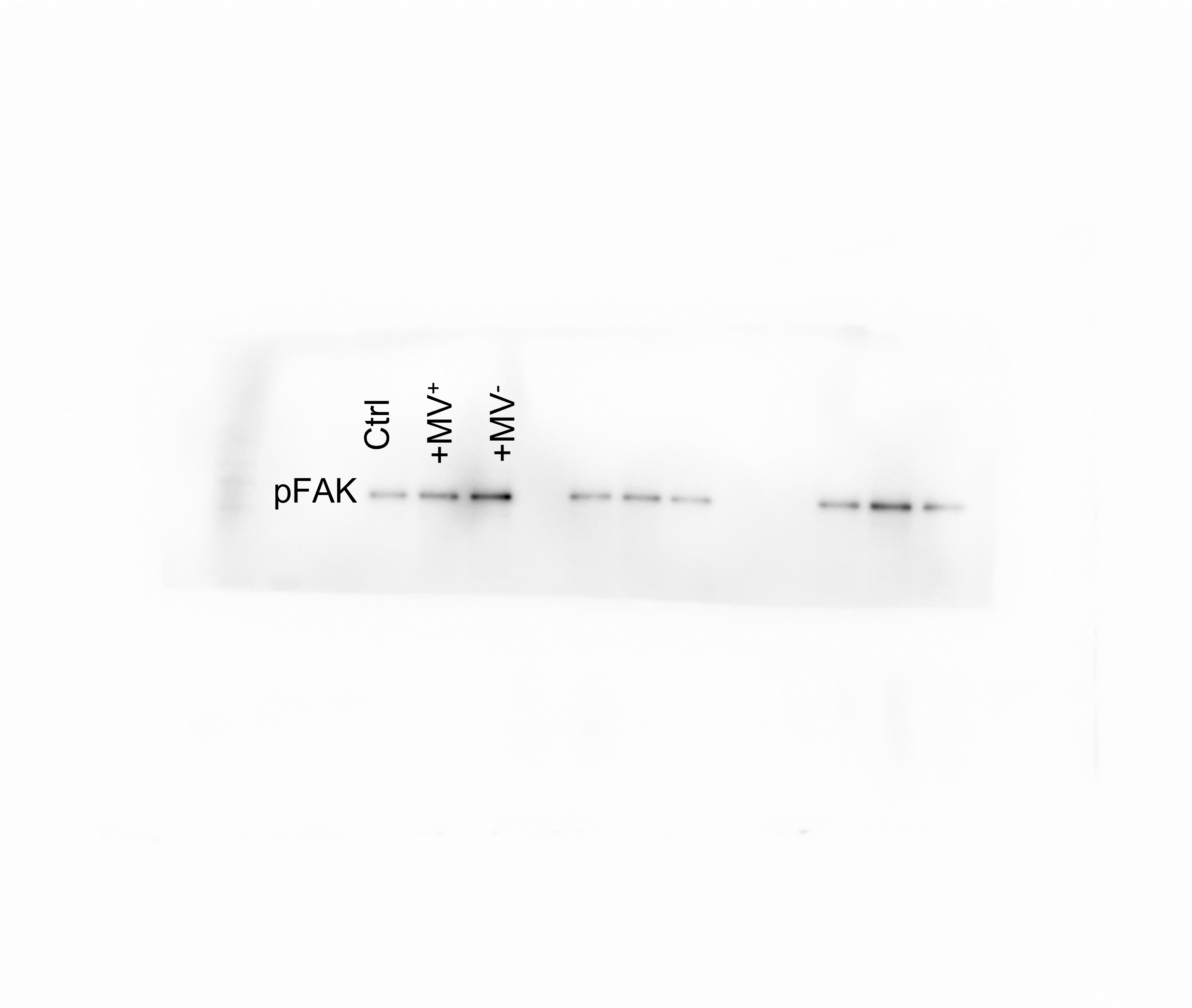

Supplement: Source data 1. [file elife-74433-data1.zip › Western Blots/Figure 2 - D - Western Blots Raw Files/Figure 2 - D pFAK.png]

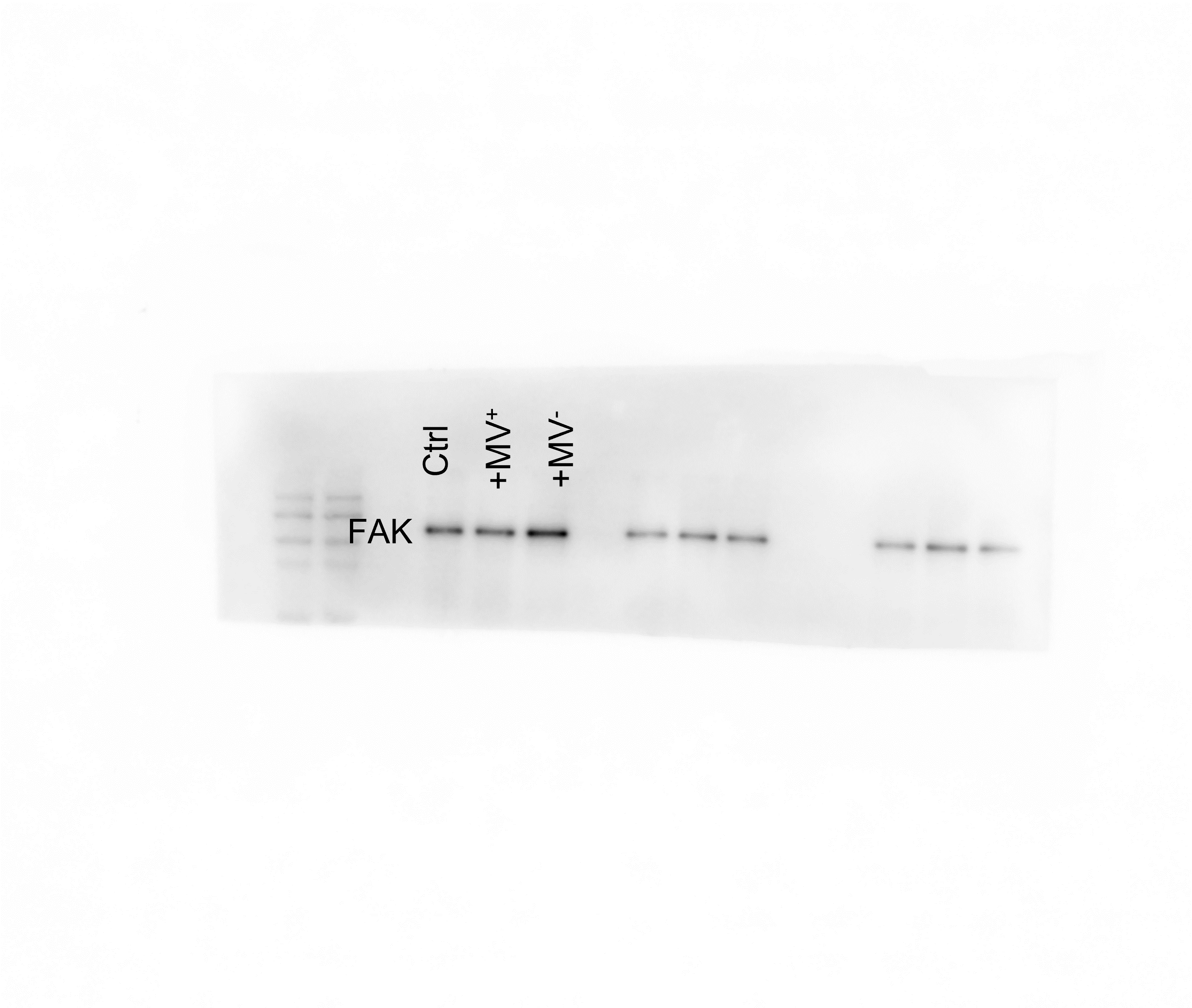

Supplement: Source data 1. [file elife-74433-data1.zip › Western Blots/Figure 2 - D - Western Blots Raw Files/Figure 2 - D FAK.png]

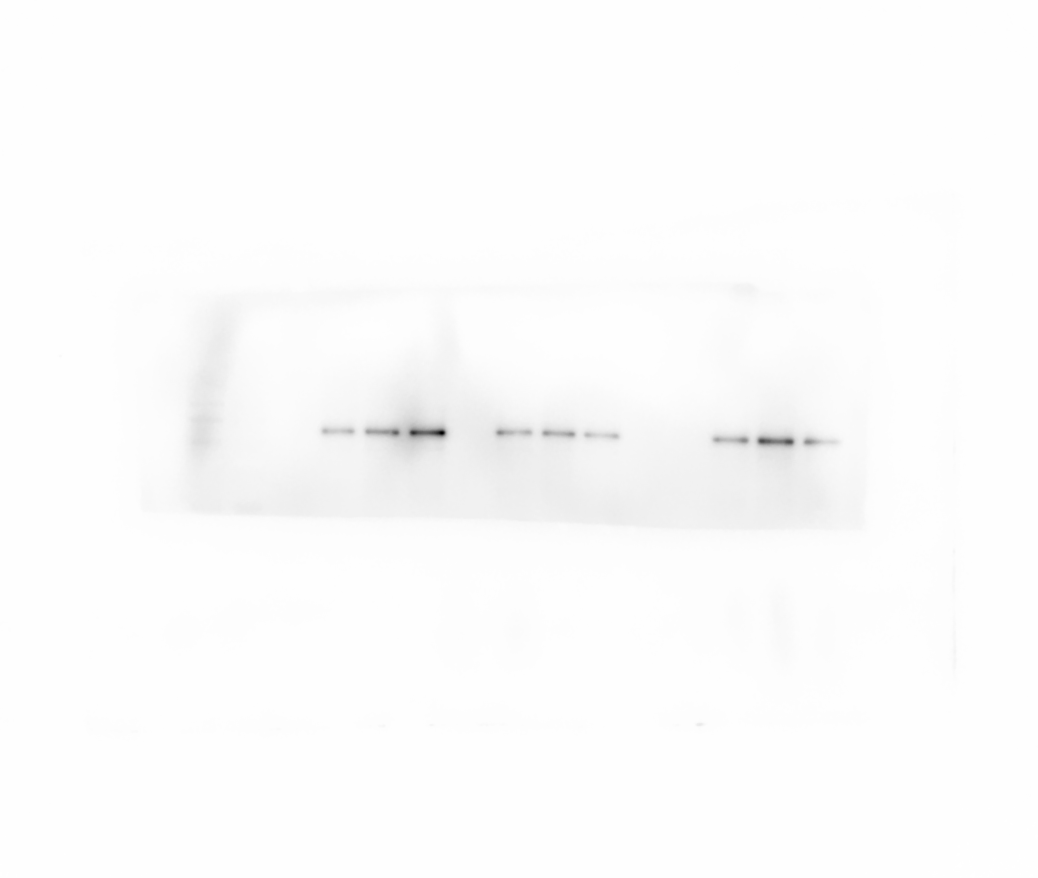

Supplement: Source data 1. [file elife-74433-data1.zip › Western Blots/Figure 2 - D - Western Blots Raw Files/Figure 2 - D - PFAK 3T3WITHMVS 052621.tif]

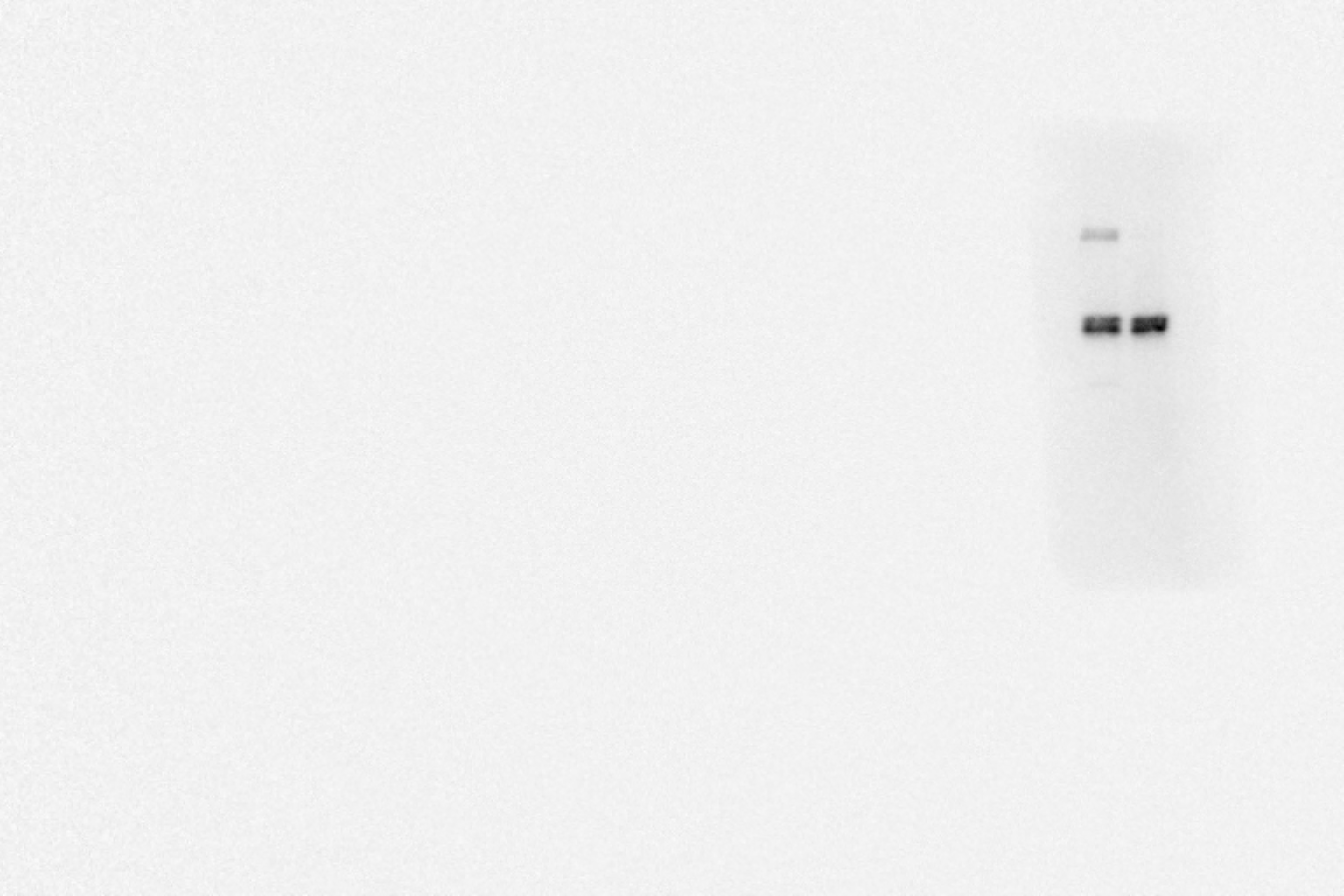

Supplement: Source data 1. [file elife-74433-data1.zip › Western Blots/Figure 4 - SuppFig 1- F - Western Blots Raw Files/Figure 3 - M - TG2 FLOT2 TG2KDINMDANONMV 042419.tif]

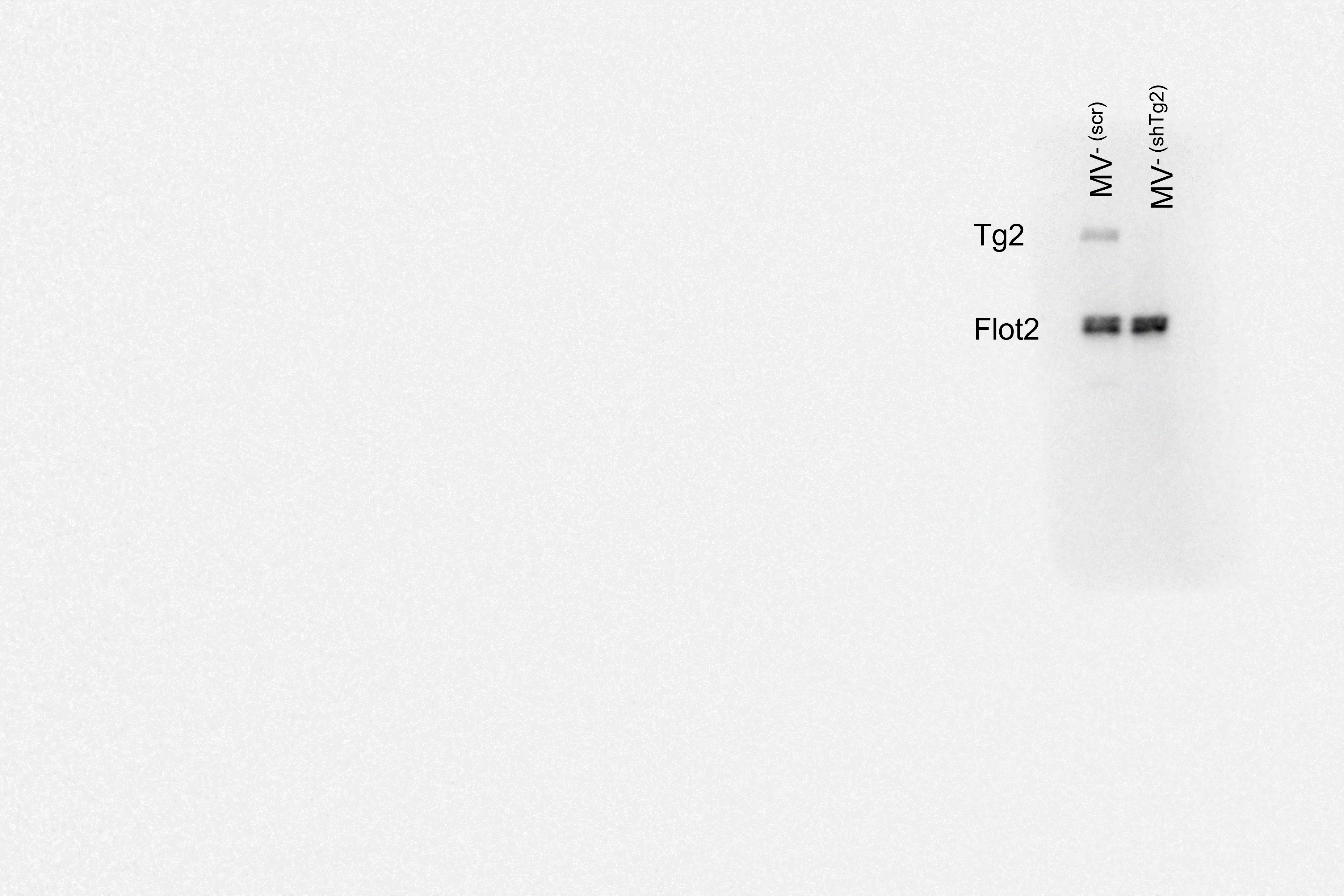

Supplement: Source data 1. [file elife-74433-data1.zip › Western Blots/Figure 4 - SuppFig 1- F - Western Blots Raw Files/Figure 3 - M Tg2 and Flot2.png]
